# Essential oils and isolated compounds for tick control: advances beyond the laboratory

**DOI:** 10.1186/s13071-023-05969-w

**Published:** 2023-11-14

**Authors:** Bruno César Ferreira Gonzaga, Mayara Macêdo Barrozo, Ana Lúcia Coutinho, Lainny Jordana Martins Pereira e Sousa, Francisca Letícia Vale, Laís Marreto, Paula Marchesini, Daniel de Castro Rodrigues, Evandro Davanço Ferreira de Souza, Gustavo Adolfo Sabatini, Lívio Martins Costa-Júnior, Lorena Lopes Ferreira, Welber Daniel Zanetti Lopes, Caio Monteiro

**Affiliations:** 1 Programa de Pós-graduação em Ciência Animal - Escola de Veterinária e Zootecnia, Universidade Federal de Goiás, Rodovia Goiânia - Nova Veneza, Km 8, Campus Samambaia, Goiânia, GO 74690-900 Brasil; 2 Faculdade de Medicina, Universidade Federal de Goiás, Campus Colemar Natal e Silva, Rua 235, s/n, Setor Leste Universitário, Goiânia, GO 74605-050 Brasil; 3 Programa de Pós-Graduação em Ciências Farmacêuticas - Faculdade de Farmácia, Universidade Federal de Goiás, Praça Universitária, no. 1166, Setor Universitário, Goiânia, GO 74605-220 Brasil; 4 MSD Saúde Animal, Avenida Dr. Chucri Zaidan, 246-96, 9º Andar, São Paulo, SP 04583-110 Brasil; 5 Ourofino Saúde Animal, Rodovia Anhanguera, Km 330, Distrito Industrial, Ribeirão Preto, SP 14140-000 Brasil; 6 Boehringer Ingelheim Animal Health, Binger Strasse 173, 55218 Ingelheim, Germany; 7 Centro de Pesquisas do CCBS, Universidade Federal do Maranhão, Avenida dos Portugueses, no. 1966, São Luís, MA 65080-805 Brasil; 8 Departamento de Medicina Veterinária Preventiva - Escola de Veterinária, Universidade Federal de Minas Gerais, Av. Antônio Carlos, no. 6627, Campus Pampulha, Belo Horizonte, MG 31270-901 Brasil; 9 Departamento de Biociências e Tecnologia - Instituto de Patologia Tropical e de Saúde Pública, Universidade Federal de Goiás-, Campus Colemar Natal e Silva - Rua 235, s/n - Setor Leste Universitário, Goiânia, GO 74605-050 Brasil

**Keywords:** Biopesticides, Botanicals, Eco-friendly, Field efficacy, Ixodidae, Tick control

## Abstract

**Background:**

Tick control is a worldwide challenge due to its resistance to acaricides. Essential oils (EOs) and isolated compounds (EOCs) are potential alternatives for tick control technologies.

**Methods:**

A review with EOs and EOCs, under field and semi-field conditions, was performed based on Scopus, Web of Science and PubMed databases. Thirty-one studies published between 1991 and 2022 were selected. The search was performed using the following keywords: "essential oil" combined with "tick," "*Ixodes*," "*Argas*," "*Rhipicephalus*," "*Amblyomma*," "*Hyalomma*," "*Dermacentor*," "*Haemaphysalis*" and "*Ornithodoros*." The words "essential oil" and "tick" were searched in the singular and plural.

**Results:**

The number of studies increased over the years. Brazil stands out with the largest number (51.6%) of publications. The most studied tick species were *Rhipicephalus microplus* (48.4%), *Ixodes scapularis* (19.4%), *Amblyomma americanum* and *R. sanguineus* sensu lato (9.7% each). Cattle (70%) and dogs (13%) were the main target animal species. Regarding the application of EOs/EOCs formulations, 74% of the studies were conducted with topical application (spray, pour-on, foam, drop) and 26% with environmental treatment (spray). Efficacy results are difficult to evaluate because of the lack of information on the methodology and standardization. The nanotechnology and combination with synthetic acaricides were reported as an alternative to enhance the efficacy of EOs/EOCs. No adverse reactions were observed in 86.6% of the studies evaluating EOs/EOCs clinical safety. Studies regarding toxicity in non-target species and residues are scarce.

**Conclusions:**

This article provides a comprehensive review on the use of EOs and EOCs to reduce tick infestations, in both the hosts and the environment. As future directions, we recommend the chemical characterization of EOs, methodology standardization, combination of EOs/EOCs with potential synergists, nanotechnology for new formulations and safety studies for target and non-target organisms, also considering the environmental friendliness.

**Graphical abstract:**

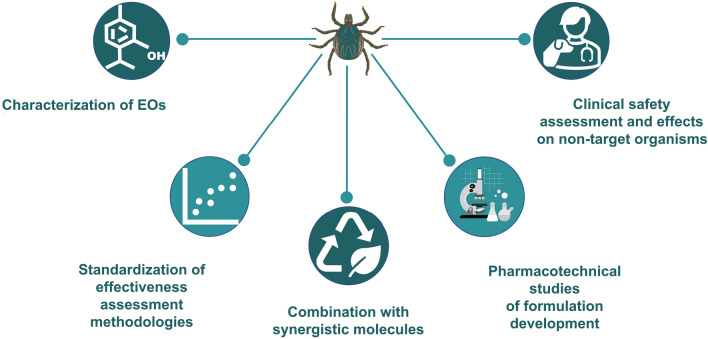

**Supplementary Information:**

The online version contains supplementary material available at 10.1186/s13071-023-05969-w.

## Background

In livestock animals such as cattle, ticks cause economic losses linked to lower body weight, live weight gain, milk production and leather quality. In addition, it leads to losses by the transmission of pathogens, mortality of cattle and costs associated with control [1, 2]. The lower productivity in herds directly impacts food production, thus representing a challenge based on the increase in world population, which is expected to reach the 9 billion mark by 2050 [3, 4].

In companion animals, ticks are responsible for blood spoliation and work as vectors of numerous pathogens (e.g. *Anaplasma platys*, *Babesia vogeli*, *B. canis, Ehrlichia canis* and *Hepatozoon canis*), impacting animal welfare and even causing death [5–7]. The increase in human population has led to a higher number of pets, as one third of families worldwide own a dog [8]. In addition, some families consider their animals as true family members because of their emotional bond [9]. Furthermore, ticks are also very important for public health, and cases of diseases generated by tick-borne pathogens in humans, such as anaplasmosis, ehrlichiosis, Lyme disease, spotted fever and tularemia, have increased considerably. Therefore, technologies to control these arthropods must be developed [10–12].

The control of ticks is mainly carried out with synthetic acaricides, composed of molecules belonging to the class of organophosphates, amidines, pyrethroids, phenylpyrazoles, macrocyclic lactones, growth inhibitors and isoxazolines [13, 14]. However, the continuous and irrational use of these drugs has resulted in tick populations resistant to almost all commercially available chemical classes. The occurrence of acaricide-resistant tick populations has been documented worldwide and for several tick species [15]. For example, there are already records of resistance for *Rhipicephalus microplus* [16–21], *R. sanguineus* s.l. [22–24], *R. annulatus* [25–29], *R. decoloratus* [30] and *Hyalomma anatolicum* [31].

**Fig. 1:**
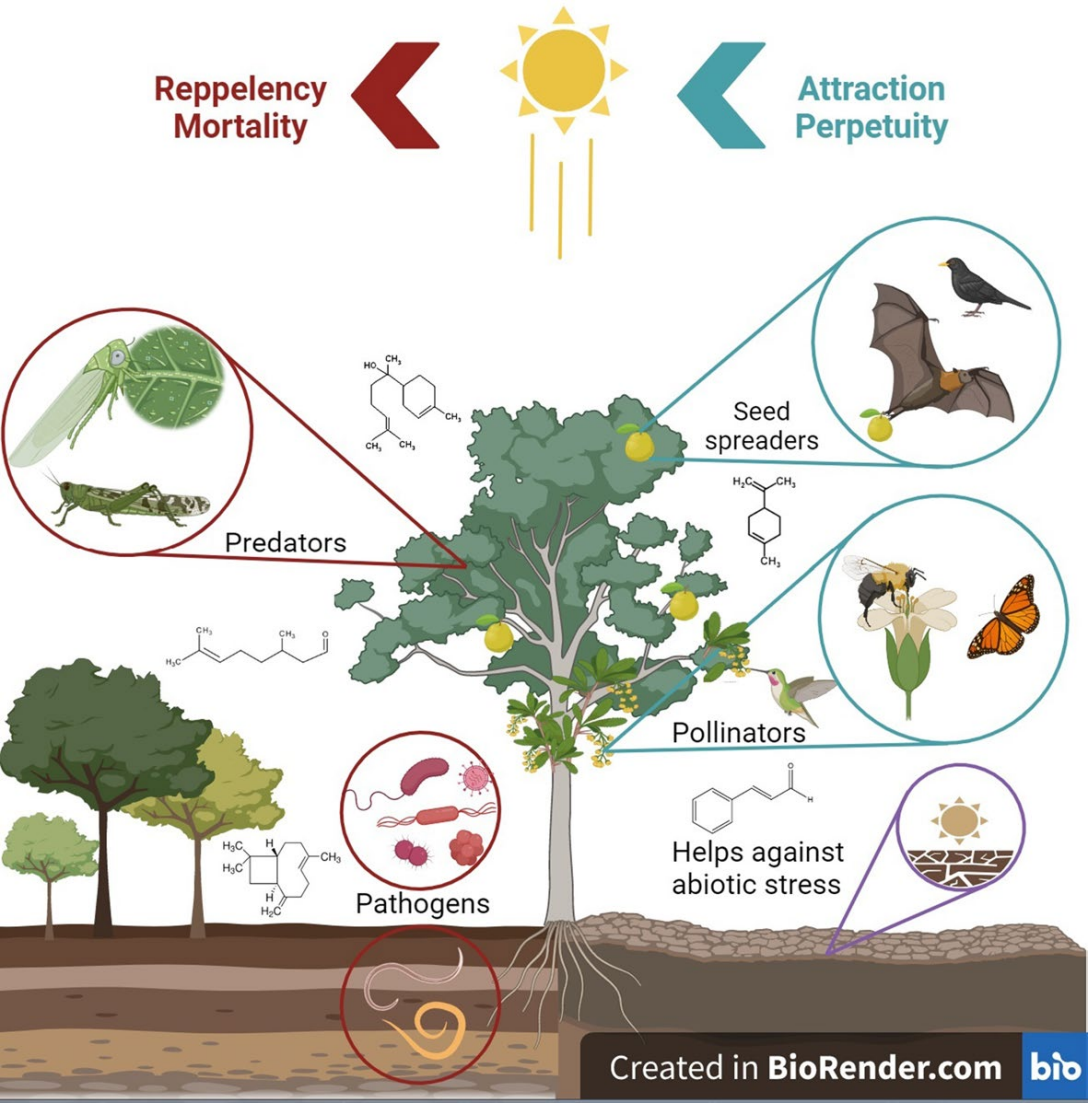
Ecological interactions of plants mediated by essential oils. Circles in green represent the attraction of seed dispersers and pollinators. Red circles represent repellency and mortality of predators and pathogens. Circle in purple represents abiotic stress factors such as dry spells

In addition to the problem of tick resistance, the consumer market has increasingly demanded pest control technologies that are eco-friendly and aligned with the concepts of “One Health” and “Sustainability.” Such aspects reinforce the development of new technologies to control these ectoparasites in a manner that is safe for humans, animals and the environment (One Health), in addition to being economically viable (Sustainability) [13, 32–34]. Essential oils (EOs) have shown potential for the development of ecofriendly acaricides [35]. These oils are natural products resulting from the secondary metabolism of aromatic plants, containing a mixture of about 20 to 60 volatile, fat-soluble and strongly odorous compounds [36]. In plants, EOs work by attracting pollinators and seed dispersers, repelling and combating parasites, pathogens and predators, in addition to assisting against abiotic stressors (Fig. 1) [35, 37, 38]. The compounds found in EOs (EOCs) can be divided mainly into two groups according to their biosynthesis: terpenes/terpenoids (such as monoterpenes and sesquiterpenes) and aromatic and aliphatic compounds, such as phenylpropanoids [36, 39].

The first studies regarding the use of EOs/EOCs for tick control [40–43] were published in the 1990s. Since then, several papers have been published demonstrating their acaricide and/or repellency activity [44–51], eventually demonstrating alterations in tick biological parameters and tissues [52–55]. Other studies provided details about the action mechanisms [56, 57] and formulation development using EOs or EOCs [58–60]. Finally, some review articles have been published on the subject [13, 61–66].

Although many studies have been produced, products on the market containing EOs or EOCs are still limited. This may be linked to the lack of studies on formulation development and efficacy evaluation under field conditions, as well as challenges related to the chemistry, manufacturing and control guidance. This review aimed to compile studies using EOs and EOCs (1991–2022) for tick control under field and semi-field conditions, presenting a critical analysis of the real state of the art of this research line, as well as suggesting priorities and directions for further studies. In addition, we present the point of view of the antiparasitic industry regarding the use of EOs and EOCs for tick control.

## Search strategy

**Fig. 2:**
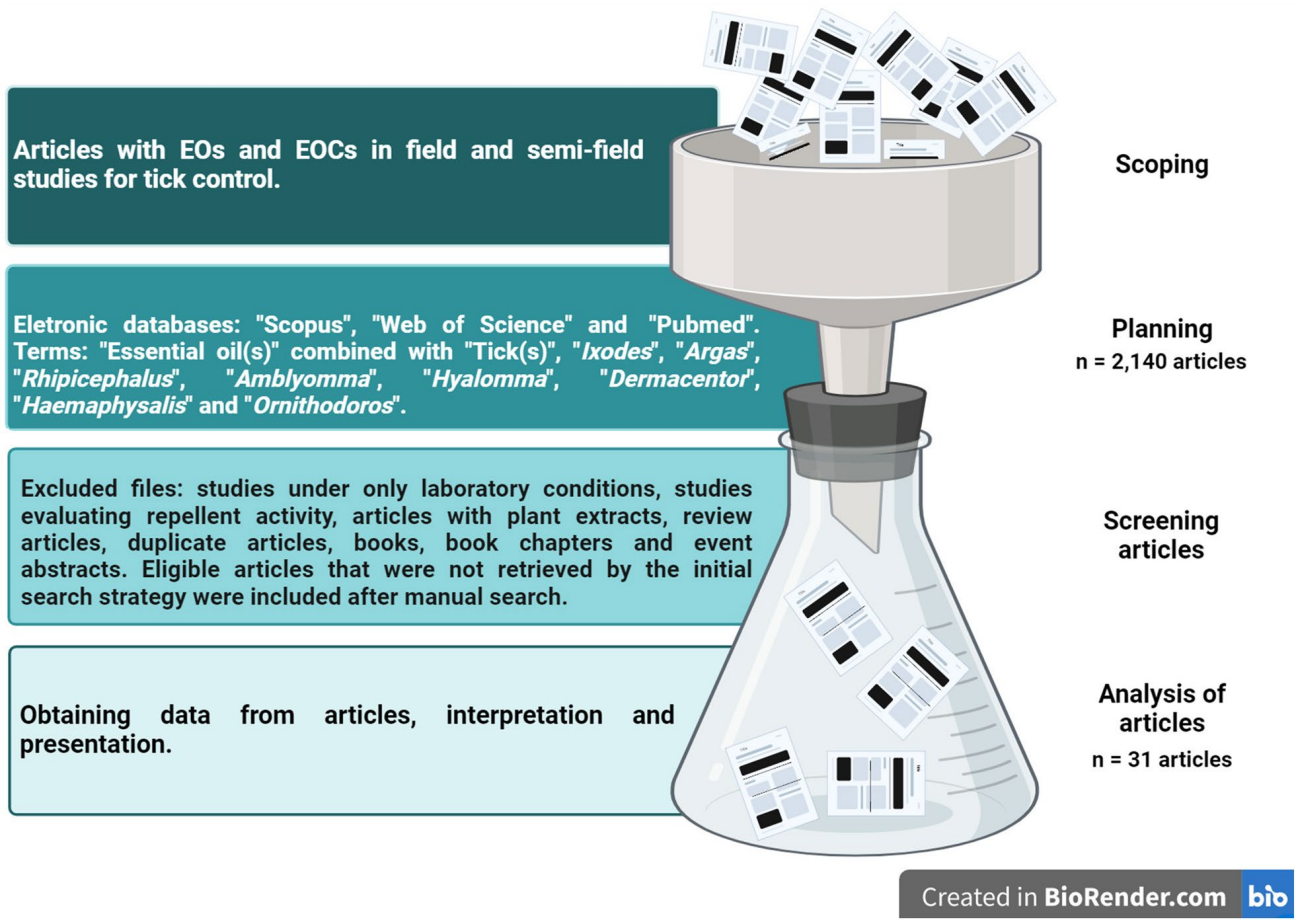
Search methodology for studies with essential oils (EOs) and essential oil compounds (EOCs) for tick control under field and semi-field conditions (*n* = 31) published from 1991 to 2022

A literature review was carried out on articles published over the last 31 years (1991–2022) by searching in the following databases: Scopus, Web of Science and PubMed. The search considered the following keywords: “essential oil” combined with “tick,” “*Ixodes*,” “*Argas*,” “*Rhipicephalus*,” “*Amblyomma*,” “*Hyalomma*,” “*Dermacentor*,” “*Haemaphysalis*” and “*Ornithodoros*.” The terms “essential oil” and “tick” were searched in both the singular and plural (Fig. 2).

The inclusion criteria considered studies that used EOs or EOCs in field and semi-field studies for tick control. The exclusion criteria considered the following situations: articles reporting studies only under laboratory conditions, studies evaluating repellent activity, articles using plant extracts, duplicate articles, review articles, books, book chapters and meeting abstracts. In addition to the authors’ expertise, a manual search process was performed by checking the list of references of the studies included in the review to identify and add eligible articles that were not retrieved by the initial search strategy. By the final search and application of the inclusion and exclusion criteria, 31 articles were selected for this review (Fig. 2).

Data from each article were compiled in a Microsoft Excel® spreadsheet, and the following parameters were evaluated: (1) year of publication; (2) country of the study; (3) type of trial; (4) number of animals; (5) host species; (6) tick species; (7) plant species used for extraction of the EOs/EOCs; (8) chemical characterization of the EOs; (9) concentration of the EOs/EOCs; (10) volume applied per animal or plot; (11) results of the acaricidal effect/efficacy on ticks; (12) clinical safety evaluation; (13) plot size; (14) evaluation in non-target organisms; (15) residue evaluation. A map showing the locations of the studies was produced using Microsoft Excel® software.

## Research with EOs and EOCs for tick control under field and semi-field conditions

### Publications per year

**Fig. 3:**
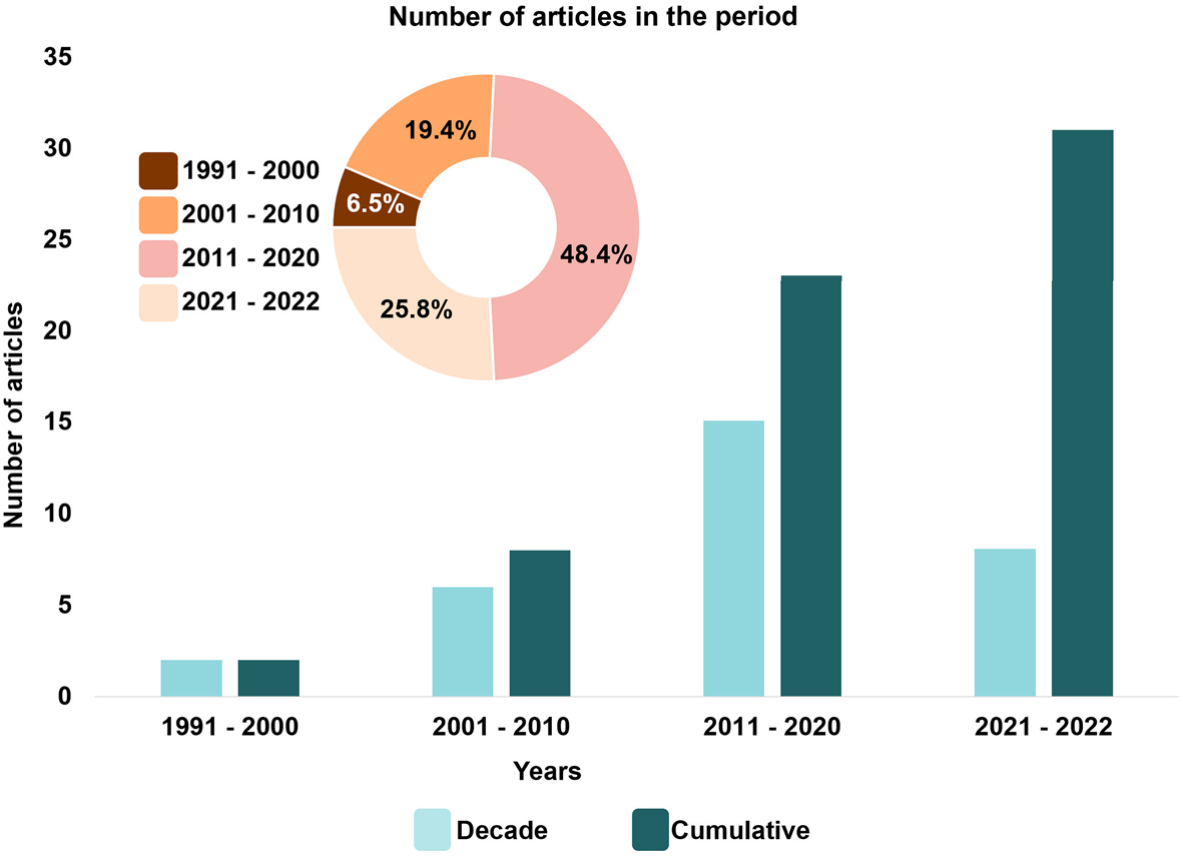
Number of publications per decade (1991–2022) using essential oils (EOs) and essential oil compounds (EOCs) for tick control under field and semi-field conditions

Thirty-one scientific articles were included in this review, with research using EOs from 19 plant species and studies using seven EOCs (Additional file 1: Figure S1, S2, S3, S4, S5 and S6). Between 1991 and 2000, only two articles (6.5%) were found, while for the following decade (2001 to 2010), six articles (19.4%) were featured. Most articles (15 publications, 48.4%) were published between 2011 and 2020, indicating a higher number of studies and greater interest in this research area. Notably, for the first 2 years of the current decade (2021–2022), eight publications were found, indicating this trend of increase in studies should remain over the next few years (Fig. 3). Such growth in the number of publications over the decades might be linked to multiple factors, including the increased number of acaricide-resistant tick populations and the need for new control technologies aligned with the concepts of One Health and Sustainability.

The number of publications worldwide has increased, thus addressing different species of ticks resistant to commercial acaricides [15], such as *Rhipicephalus microplus* [16–18, 21, 30, 67–75], *R. sanguineus* s.l. [22–24, 76–79], *R. annulatus* [59], *R. australis* [80], *H. anatolicum* [31], *R*. *appendiculatus*, *R*. *bursa*, *R. decoloratus*, *R*. *evertsi*, *Amblyomma mixtum* and *A*. *hebraeum* [81].

### Publications by country

**Fig. 4:**
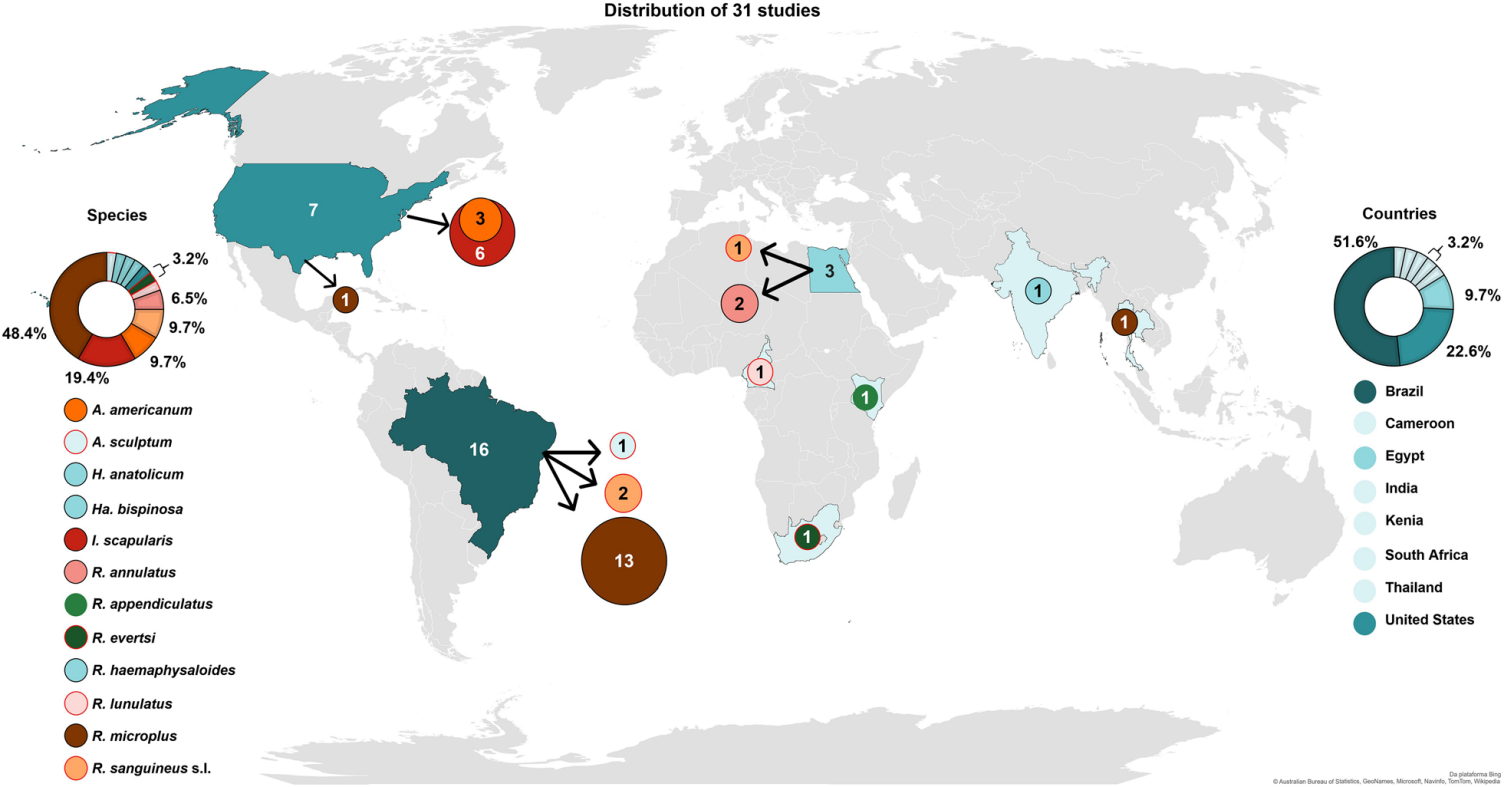
Geographic distribution of 31 field and semi-field studies using essential oils (EOs) or essential oil compounds (EOCs) for tick control between 1991 and 2022. Colored circles, with sizes proportional to the number of studies, represent different species of ticks. Abbreviation of tick genera: *Amblyomma* (*A*.); *Ixodes* (*I*.); *Hyalomma* (*H*.); *Haemaphysalis* (*Ha*.); *Rhipicephalus* (*R*.). Source: adapted from the Bing platform, 2022

The research studies using EOs and EOCs in both field and semi-field conditions were conducted in eight countries, in the following order: Brazil (51.6%), the US (22.6%) and Egypt (9.7%) (Fig. 4). The greater representation of Brazil might be linked to multiple factors, including the particular severity of ticks regarding the livestock industry and animal health in the country, the huge plant diversity and culture of using natural products for health issues and the interest of some Brazilian researchers. Brazil has the largest commercial cattle herd in the world (224.6 million cattle) [82], being one of the biggest producers of beef (2.975 million tons per year) [83] and the fourth largest producer of milk [84] (35.3 billion liters per year) [82]. In addition, Brazil has the second largest dog population in the world (estimated at 58.1 million dogs) and takes in the sixth highest revenue in the global pet market [85, 86]. Therefore, there is a great demand for new technologies to control ticks on livestock and dogs in this country. Regarding plant biodiversity, Brazil has the largest number of described species in the world (55% of endemic terrestrial plant species) [87], hence the great biodiversity of raw material for studies on the activities of botanical compounds on ticks.

### Publications by tick species

*Rhipicephalus microplus* (48.4%) was the most studied tick species, followed by *I. scapularis* (19.4%), *A. americanum* and *R. sanguineus* s.l. (9.7% each) and *R. annulatus* (6.5%). Research was also conducted with *Rhipicephalus lunulatus*, *R. evertsi*, *R. appendiculatus*, *Amblyomma sculptum*, *H. anatolicum*, *Haemaphysalis bispinosa* and *R. haemaphysaloides* (3.2% = one study with each species). All studies targeted ticks of economic or public health importance (Fig. 4, Tables 1, 2, 3).

**Table 1:** General data (treatments and efficacy) compiled from 16 articles using essential oils (EOs) and essential oil compounds (EOCs) to control *Rhipicephalus microplus* and *R. annulatus* in cattle studies from 1991 to 2022

Tick (stage)	EOs/EOCs	Concentration	Volume and form of application	No. of animals/treated group	Type of infestation	Main results	References
*R. microplus* (A)	*Cymbopogon citratus* and C*. nardus C. winteranius Jowitt* (major compounds: not mentioned)	…	… (Spray)	…	Field trial Natural	Semi-engorged females collected (after spraying) died 48 h after EO application and engorged females did not oviposit	Chungsamarnyart and Jiwaginda [183]
*Rhipicephalus microplus* (A)	*C. winteranius Jowitt* (major compounds: not mentioned)	10% and 100%	40 mL (pour-on); 2 L (spray)	15	Field trial Artificial	EO significantly reduced the number of ticks	Martins and González [124]
*R. microplus* (L, N, A)	*C. nardus* (major compounds: citronellal 50.07%, geraniol 13.87%, citronellol 7.93%)	4%	3 L each 7 days for 28 days (Backpack Sprayer)	5	Field trial Natural	Efficacy of 35.3%, 11.8%, 34% and 42.4% at days 7, 14, 21 and 28 after treatment	Agnolin et al. [157]
*R. microplus* (N, A)	*C. nardus* (major compounds: citronellal 50.07%, geraniol 13.87%, citronellol 7.93%)	3% and 4%	3 L, single treatment (Backpack sprayer)	5	Field trial Natural	Efficacy mean of 22.5 and 39.1% at concentrations of 3 and 4%, respectively, 28 days after treatment	Agnolin et al. [184]
*R. microplus* (L, N, A)	*Co. citriodora* (major compounds: citronellal 70.4%, isopulegol 16.3% and citronellol 5.5%)	3.5%	4 L (backpack sprayer)	6	Field trial Natural	Efficacy mean of 96.4%, 21 days after treatment	Olivo et al. [159]
*R. microplus* (L, N, A)	*Tagetes minuta* (rich in terpenes)	20%	50 mL (pour-on)	6	Pen trial Artificial	Efficacy of 99.98%	Andreotti et al. [140]
*R. microplus* (L, N, A)	*C. winterianus* (major compounds: not mentioned)	8.6%	4 L, single treatment (Backpack sprayer)	6	Field trial Natural	Efficacy mean of 90%, 21 days after treatment	Agnolin et al. [141]
*R. microplus* (L, N, A)	Original oil of *Co. citriodora* and modified oil of *Co. citriodora* (N-prop-2-inylcitronellylamine)	1.5% and 1.5%	… …	6	Field trial Natural	Treatment with EO (original and formulated) did not significantly reduce the number of ticks	Chagas et al. [185]
*R. microplus* (L, N, A)	*Melaleuca alternifolia* (major compounds: terpinen-4-ol 41.98%, γ-terpinene 20.15%)	0.75% (nano particle) and 5% (unformulated)	400 mL, single treatment (spray)	5	Field trial Natural	The unformulated EO had a greater effect on adults. Encapsulated nano EO had a greater effect on biology of ticks (34.5% efficacy)	Boito et al. [125]
*R. microplus* (L, N, A)	*Cinnamomum* sp. (major compound: cinnamaldehyde 41.27%)	0.5% (nanocapsules); 0.5% (nanoemulsion) and 5% (unformulated)	50 mL, single treatment (neck, legs, ventral and inguinal region)	4	Field trial Natural	Efficacy was 90.5%; 100% and 63.5% in treatments with unformulated EO, nanocapsules and nanoemulsion, respectively	Santos et al. [126]
*R. microplus* (L, N, A)	Eugenol	5%	10 mL/100 kg, single treatment (pour-on)	6	Pen trial Artificial	Efficacy mean of 13.80%, 20 days after treatment	Valente et al. [158]
*R. annulatus* (L, N, A)	Thymol + deltamethrin Thymol + *E.globulus* + deltamethrin *E. globulus* (major compounds: not mentioned)	5%	… treated twice, 2-week interval (spray)	5	Field trial Natural	Efficacy mean of 88.33 and 95% for thymol + deltamethrin and thymol + *E. globulus* + deltamethrin. Engorged females deposited small egg masses unable to hatch	Arafa et al. [59]
*R. microplus* (L, N, A)	Essentria® IC-3 (rosemary oil 10%, geraniol 5% and peppermint oil 2%) EOs—major compounds: not mentioned	6.25%	7.5 L, single treatment (spray race)	4	Pen trial Artificial	Less engorged females recovered from the treated group for 21 days. Considering the biological parameters of ticks, the efficacy was 70%	Klafke et al. [142]
*R. microplus* (L, N, A)	(*E*)-cinnamaldehyde	0.1%	5 L, single treatment (backpack sprayer)	10	Field trial Natural	The animals showed signs of intoxication, such as sialorrhea and muscle tremors. The experiment was interrupted	Gonzaga et al. [21]
*R. microplus* (L, N, A)	*Lippia sidoides* (major compound: thymol 40.3%, p-cymene 17.2%, *E*-caryophyllene 8.99%)	1%	3 L, single treatment (backpack sprayer)	10	Field trial Natural	The efficacy range between day 3 to 28 after treatment was 23.3 to 63.2%	Pereira et al. [180]
*R. annulatus* (L, N, A)	*Pelargonium graveolens* (major compound: citronellol 14.44%, geraniol 11.08%, linalool 7.74%, citronellyl 7.66%)	10% nanoemulsion Combination with sesame oil	400 mL, single treatment (spray)	5	Field trial Natural	Efficacy mean of 87.97% and 74.83% for and *Pelargonium graveolens* nanoemulsion (nano) and *P. graveolens* + sesame oil. Females treated with *P. graveolens* (nano) did not oviposit	Ibrahium et al. [143]

Abbreviations of tick genera: *Rhipicephalus* (*R*.)

Abbreviations of the stages of ticks: larva (L); nymph (N); adult (A)

Abbreviations of the genera of plants: *Cymbopogon* (C.), *Corymbia (Co.), Tagetes (T.), Melaleuca (M.), Eucalyptus (E.), Lippia (L), Pelargonium (P.)*

…—Information not mentioned

**Table 2:** General data (treatments and efficacy) compiled from seven articles using essential oils (EOs) and essential oil compounds (EOCs) for tick control in studies with dogs, rabbits, sheep and goats from 1991 to 2022

Tick (stage)	Host	EOs/EOCs	Concentration	Volume and form of application	No. of animals/treated group	Type of infestation	Main results	References
*Rhipicephalus appendiculatus* (L, N, A)	Rabbits	*Ocimum suave* (major compound: not mentioned)	2, 5 and 10%	5 mL per ear (topical spray)	9	Artificial	Mortality of 100 for larvae and nymphs and 74.5% for adults at a concentration of 10%	Mwangi et al. [135]
*Hyalomma anatolicum*; *Haemaphysalis bispinosa*; *Rhipicephalus haemaphysaloides* (–- – “tick count”)	Goats	*Cymbopogon citratus* and *C. nardus* (major compound: not mentioned)	25, 33 and 50%	…, single treatment (spray)	6	Natural	Lemongrass oil eliminated all ticks in 24 h and citronella oil in 48 h	John et al. [132]
*Rhipicephalus evertsi* (N, A)	Sheep	*Th. trilobata* (major compound: alpha-pinene 21.6%, alpha-phellendrene 21%, limonene 12.8%)	5 mg/mL and 10 mg/mL	2 drops in the attachment site of the tick (ear, genital/anal areas)	1	Natural	Mortality of 100% (n = 7) of ticks at a concentration of 10 mg/mL	Peebles et al. [134]
*Rhipicephalus lunulatus* (L, N, A)	Goats	*Ch. ambrosioides* (major compound: not mentioned)	0.06, 0.09 and 0.12 µL/g	Soap (foam) twice a day, focusing on points where ticks are	10	Natural	Mortality (cumulative) after the 8th day was 76.12, 90.27 and 96.29%, at concentrations of 0.06, 0.09 and 0.12 µL/g, respectively	Kouam et al. [133]
*R. sanguineus* sensu lato (L, N, A)	Dogs	*T. minuta* (major compound: not mentioned)	20%	20 µL (topical spray)	5	Artificial	The EO resulted in 100% efficacy, the ticks died 24 h after application	Silva et al. [130]
*R. sanguineus* sensu lato (A)	Dogs	Lacecca® (Allicin + *A. sativum* + *B. napus)*	0.05 + 2.5 + 8%	0.25 mL/kg for 3 days (oral spray)	10	Artificial	EO resulted in 100% efficacy in preventing infestation by *R. sanguineus* s.l. and a treatment efficacy of 75 to 99% from the first to the third dose	Amer and Amer [131]
*R. sanguineus* sensu lato (L, N, A)	Dogs	Thymol + eugenol	5 + 5 mg/mL (0.50% (p/p)	10 mL/kg (topical spray)	5	Artificial	The nanoemulsion reduced the number of larvae on the animals and affected the reproductive parameters of engorged females (percent control = 85%)	Monteiro et al. [60]

Abbreviations of tick genera: *Rhipicephalus* (*R*.); *Hyalomma* (*H*.); *Haemaphysalis* (*Ha*.)

Abbreviations of the stages of ticks: larva (L); nymph (N); adult (A)

Abbreviations of the genera of plants: *Cymbopogon* (C.)*, Ocimum (O.), Thelechitonia (Th.), Tagetes (T.), Chenopodium (Ch.), Allium (A.), Brassica (B.)*

…—Information not mentioned

**Table 3:** Treatment and efficacy compiled from eight articles using essential oils (EOs) and their compounds (EOCs) for tick control in environmental studies from 1991 to 2022

Tick (stage)	EOs/EOCs	Type of infestation	Concentration	Plot size	Main results	Reference
*I. scapularis* and *A. americanum* (N, A)	Nootkatone; Nootkatone nanoemulsion and Carvacrol	Naturally infested environment	1; 2; 5%; 3.1% (nanoemulsion) and 0.05; 5%	100 m^2^	Two compounds (5%) were able to suppress 100% the nymphs of *I. scapularis* and *A. americanum* for 2 days, maintaining an efficacy > 65% up to 21 days after application	Dolan et al. [150]
*I. scapularis* (L, N, A)	Eco-Exempt IC2 (10% rosemary oil, 5% geraniol, 2% peppermint oil)	Naturally infested environment	3.1%	10,000 m^2^ (L, N) and 100 m^2^ (A)	Efficacy of 100%, 2 weeks after application. In larvae, the control was 63.1% (5 weeks post spray). For adults, the control was 93.8% in the 14th week post spray	Rand et al. [186]
*I. scapularis* and *A. americanum* (N)	Nootkatone	Naturally infested environment	2%	100 m^2^	Efficacy of 96.5% for *I. scapularis* and 91.9% for *A. americanum,* after 42 and 35 days, respectively	Jordan et al. [146]
	Eco Trol T&O (10% rosemary oil, 2% peppermint oil and 0.5% sodium lauryl sulfate, with wintergreen oil, vanillin, lecithin and butyl lactate)		46.9 mL/L and 78.1 mL/L	100 m^2^	The applications resulted in 90.8 and 87.3% control of *I. scapularis* and *A. americanum*, respectively	
	Carvacrol		2%	100 m^2^	The applications resulted in 92.3% and 92.9% control of *I. scapularis* and *A. americanum*, respectively	
*Ixodes scapularis* Say (N)	Nootkatone	Naturally infested environment	0.46–0.84%	150–387 m^2^	Efficacy of 100% for *I. scapularis* nymphs after 3 days and 49% after 16 days. With lignin encapsulation, the efficacy was 100% throughout during 56 days	Bharadwaj et al. [147]
*Ixodes scapularis* Say (L, N, A)	Eco-Exempt IC2 (10% rosemary oil, 5% geraniol, 2% peppermint oil)	Naturally infested environment	3.1%	100 m^2^	IC-2 was as effective as bifenthrin (pesticide) in L, N and A and was less toxic in non-target species	Elias et al. [167]
*R. microplus* (L)	Crystals of thymol	Experimentally infested environment	2.5; 5; 10; 15 and 20 mg/mL	0.05 m^2^	At the highest concentrations (10, 15 and 20 mg/mL) the number of live larvae decreased by > 95% compared to the control group	Araújo et al. [148]
*A. sculptum* (L)	Thymol + eugenol and carvacrol + eugenol	Experimentally infested environment	5 mg/mL	0.03 m^2^	Efficacy in treatments with thymol + eugenol and carvacrol + eugenol was 63 and 42%, respectively	Vale et al. [152]
*I. scapularis* and *A. americanum* (N, A)	Essentria® IC-3 (10% rosemary oil, 5% geraniol, 2% peppermint oil)	Naturally infested environment	86.6 mL active ingredient/plot	100 m^2^	First application (April)—Efficacy exceeding 90% for 3 weeks for nymphs; second application (May)—efficacy of 100% and ≥ 90% for *I. scapularis* and *A. americanum* nymphs for another 3 and 2 weeks, respectively. For adults, efficacy was low	Schulze and Jordan [149]

Abbreviations of tick genera: *Ixodes* (*I*.), *Amblyomma* (*A*.); *Rhipicephalus* (*R*.)

Abbreviations of the stages of ticks: larva (L); nymph (N); adult (A)

*Rhipicephalus microplus* (cattle tick) was the most studied tick species, probably because of its wide geographic distribution and great economic importance for the cattle industry worldwide [88]. The annual losses attributed to this tick in Brazil and Mexico were estimated at $3.24 billion and $573.61 million, respectively [2, 89]. In addition, it is notable that most of the tick control products available on the market show low efficacy in controlling this tick [16, 71, 90] based on the increasing records of resistant populations, especially multidrug-resistant populations, in Central American, South American and Asian countries [15, 17, 18, 68–70, 73, 91, 92].

*Ixodes scapularis* (black-legged tick) was the second most studied species, followed by *A. americanum* (lone star tick) and *R. sanguineus* s.l. (brown dog tick). These first two species have great public health importance in the US as vectors of disease agents to humans [93]. The black-legged tick is the vector of the causative agents of diseases such as Lyme disease (*Borrelia burgdorferi*), human granulocytic anaplasmosis (*Anaplasma phagocytophilum*) and babesiosis (*Babesia microti*). The lone star tick is the vector of *Ehrlichia chaffeensis* (human monocytic ehrlichiosis) and *E. ewingii* (human granulocytic ehrlichiosis), *Borrelia lonestari* (tick-associated rash illness) and *Francisella tularensis* (tularemia) [12, 94, 95].

Brown dog ticks have great importance in animal health as vectors of several pathogens for dogs, such as *E. canis*, *B. vogeli*, *Mycoplasma haemocanis* and *H. canis* [96]. In addition, *R. sanguineus* s.l. can also parasitize humans and act as vectors of *Rickettsia conorii* and *R. rickettsii*, among other disease agents [97–102]. Notably, resistance of *R. sanguineus* s.l. has also been described in pyrethroids, amidines, organophosphates, phenylpyrazoles and macrocyclic lactones [22–24, 78].

Despite also being of veterinary and public health concern, the other above-mentioned ticks are more geographically limited. For example, *R. annulatus* (North American Texas fever tick) is most prevalent in the Mediterranean region and has been eradicated from the US [103, 104]. The ticks *R. lunulatus*, *R. e. evertsi* (red-legged tick) and *R. appendiculatus* (brown ear tick) are found in Africa parasitizing livestock (horses, cattle, goats and sheep) and wildlife animals, such as African buffaloes and antelopes, causing morbidity and mortality in these animals [105]. In Brazil, *A. sculptum* is a tick that has capybaras, horses and tapirs as primary hosts but can accidentally feed on humans and transmit *R. ricketsii* [106]. Finally, *H. anatolicum*, *Ha. bispinosa* and *R. haemaphysaloides* are common in India (Asia) parasitizing small ruminants and horses [107].

### Publications by compounds tested and chemical characterization of essential oils

**Fig. 5:**
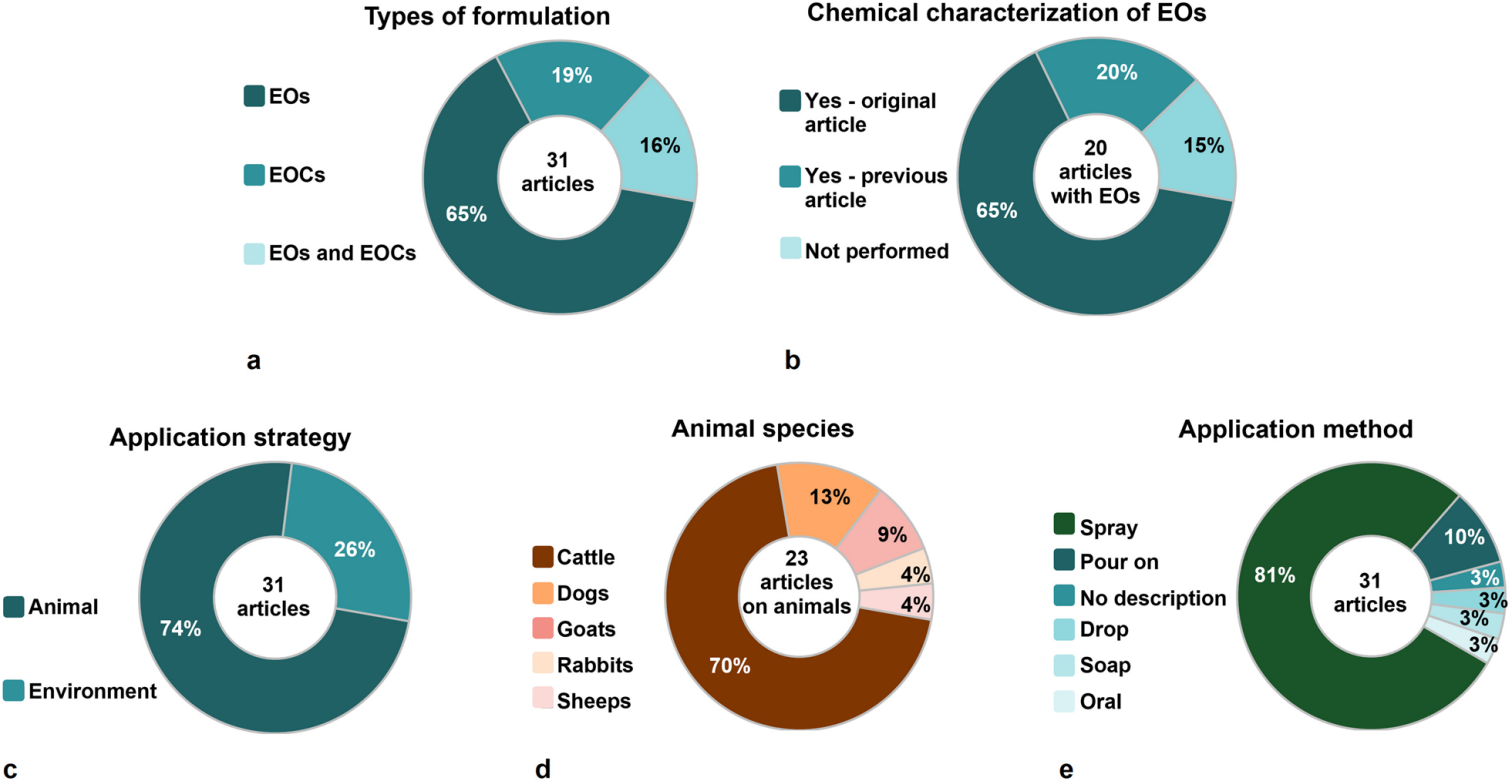
Field and semi-field studies (*n* = 31) using essential oils (EOs) and essential oil compounds (EOCs) for tick control in the period 1991–2022. **a** Percentage of studies with EOs and EOCs. **b** Percentage of studies that performed chemical characterization of EOs or not; **c** percentage of EOs and EOCs application strategies; **d** percentage of animal species used in studies with EOs/EOCs; **e** percentage of methods for applying EOs/EOCs to animals and the environment

Most field and semi-field studies used EOs (65%), followed by EOCs (19%) and both EOs and EOCs in 16% (Fig. 5a, Tables 1, 2, 3). The chemical composition was evaluated in 85% of the studies that used EOs; however, in some cases (20%), the characterization was not performed in the study itself but in a previous study conducted by the same research group. In 15% of these studies, chemical characterization of the EOs was not performed (Fig. 5b). Studies using commercial products containing EOs were not considered in this analysis.

It is known that the same plant species can present varied compositions and acaricidal activity according to the genotype, soil, collection site, time of year, harvest year, plant part used, extraction method and storage conditions [108–111]. It has been shown that EOs from the same plant species have differences in acaricidal activities due to variations in chemical composition [111–114]. Therefore, the chemical characterization of EOs is a fundamental aspect of identifying their active compounds.

There are advantages and disadvantages to using EOs and EOCs. As an advantage, EOs present lower toxicity to vertebrates compared to the major compounds isolated from them when tested alone [115]. Furthermore, their mixtures can result in synergistic effects due to the presence of compounds with different action mechanisms [35, 37]. As a negative aspect, EOs present variations in chemical composition, which can hinder commercial applications due to the lack of standardization, hence generating difficulties in quality control and obtaining raw materials on a large scale. A potential solution would be to work with marker compounds (putatively the active principles), including predetermined amounts of a key compound that can guarantee efficacy against ticks; however, it is not a simple task [116]. The EOCs have the advantage of standardization and the ease of obtaining the active ingredient on a large scale for developing commercial formulations. However, the use of compounds isolated from the EOs can raise the toxicity of the formulation for animals [115], as already demonstrated in guinea pig using (*E*)-cinnamaldehyde, a major compound found in cinnamon EO [21].

### Experimental design: animal species, number of animals used and administration of the formulations on hosts and the environment

Variations occurred regarding the animal species, number of animals per treated group used in the experiments, volume of formulation applied to the animals and forms of application. Among the 31 field and semi-field studies using EOs and EOCs for tick control, 74% applied the formulations on the animals (cattle, dogs, goats, sheep and rabbits) (Fig. 5c and d, Tables 1 and 2), while 26% applied them in the environment (Fig. 5c, Table 3).

#### Species and number of animals used

Among the 23 studies using hosts, 70% (16/23) used cattle, 13% (3/23) dogs, 9% (2/23) goats, 4% (1/23) sheep and 4% (1/23) rabbits. The number of animals per treated group varied from 4 to 15 for cattle, 5 to 10 for dogs, 6 to 10 for goats, 1 for sheep and 9 for rabbits (Fig. 5d, Tables 1 and 2).

In this regard, the first version of the guidelines of the World Association for the Advancement of Veterinary (WAAVP) for evaluating the efficacy of acaricides against ticks of ruminants recommended a minimum of three animals per group [117]. The new guidelines, published in 2022, recommended a minimum of 20 animals per treated group [118]. These recommendations may vary regionally. As an example, Brazilian legislation recommends the use of 10 animals per group [119]. For dogs, the WAAVP recommends a minimum of six animals per group [120, 121].

Animal experimentation with a larger number of animals poses a challenge for conducting research under field and semi-field conditions due to cost and ethical issues. Thus, there must be efforts to find alternatives to such a challenge by respecting the principles of the 3Rs (replacement, reduction and refinement) of animal research [122]. In this sense, it is also important to develop tests in animal models, predictive tests, computational modeling and validation alternatives for formulations developed with EOs and EOCs [123].

#### Formulations and administration on hosts

The formulations and administration routes of EOs/EOCs most used on hosts, topical spray (81%) was the most frequent, followed by pour-on (10%), drop on the tick attachment site (3%), soap foam (3%) and oral spray (3%). One publication did not describe the administration route (Fig. 5e, Tables 1 and 2).

In the publications with cattle using spray formulations, in 53% of the studies, at least three liters of solution was applied per animal (Table 1). As to the volume of formulation, Martins and González [124] used 2 L of solution in experimentally infested cattle weighing 350 kg, while Boito et al. [125] and Santos et al. [126] used only 400 mL and 50 mL, respectively, in naturally infested adult cattle. However, Santos et al. [126] performed the application only on the neck, legs, and inguinal and ventral regions of these bovines. Some of the studies did not report the formulation or volume applied, the age category (young, adult) or the weight of the animals. For spray commercial acaricides, it is recommended to treat the whole body of the bovine using 4–5 L (1 L per 100 kg animal), whereas, for pour-on formulations, the dosage varies according to the animal body weight and the dorsal line of the animal is treated [16, 127–129].

For dogs, two studies used a topical non-commercial formulation [60, 130] and one used an oral commercial formulation [131]. All works used experimental tick infestations (Table 2). Silva et al. [130] released the ticks in a chamber (5 × 3 cm) glued on the back of the dogs. The area of the chamber was sprayed once with 20 µL wild marigold (*Tagetes minuta*) EO after 24 h of tick infestation. Monteiro et al. [60] released the ticks on the dog’s nape and the dogs were secured for 10 min to allow tick distribution. After 24 h, the whole body of the dogs was sprayed once with thymol + eugenol EOCs microemulsion (10 mL/kg). In turn, Amer and Amer [131] sedated the animals and released the ticks on the dogs’ fur of the back, lateral side and head. The dogs received the spray oral treatment with Lacecca® (garlic oil 2.5%, allicin 0.05%, rapeseed oil 8%) for 3 successive days/month, before or after the tick infestation, depending on the group, at the dosage of 0.25 mL/kg.

For small ruminants (Table 2), soap foams of mastruz (*Chenopodium ambrosioides*) EO and spray lemongrass EO (*Cymbopogon citratus* and *C. nardus*) were used on naturally infested goats against different tick species [132, 133]. Three concentrations of soap with *C. ambrosioides* EO were developed, and the soap foam was applied on the goats, twice a day (morning and evening), focusing on points where *R. lunulatus* were present [133]. In turn, drops of wedelia (*Thelechitonia trilobata*) EO were applied on *R. e. evertsi* attachment sites in naturally infested sheep [134]. In addition to their differences in administration, it is impractical to use foam and drops at the site of tick attachment as a management routine on extensive farming of small ruminants as, once attached, ticks that parasitized goats and sheep, such as *R. e. evertsii* and *R. lunulatus*, prefer to feed mainly inside ears and tail (near genital/anal region). The work developed in rabbits (New Zealand) held in cages was developed with an experimental infestation on the rabbit ears using a cotton bag [135]. The alfavaca (*Ocimum suave*) EO was sprayed 5 mL per ear on the 2nd day of *R. appendiculatus* feeding using a laboratory animal, which is not with the preferred host. In addition, there are no studies in the same scenario for comparison and discussion.

#### Administration of the formulations in the environment

Eight studies using EOs/EOCs for tick control in the environment (field or semi-field conditions) were evaluated (Table 3). The studies were performed in either naturally or experimentally infested areas covering different plot sizes using spray formulations. The targeted ticks were those of public health importance (*I. scapularis*, *A. americanum* and *A. sculptum*), whose main hosts are usually wild animals. Managing wild animals is known to be difficult, thus requiring adopting different measures, such as applying acaricides in the environment.

For example, in endemic and risk areas for spotted fever rickettsiosis in Brazil, *A. sculptum* populations are usually maintained by capybaras (*Hydrochoerus hydrochaeris*). The application of acaricides on capybaras may not be an easy task and the fact that the animals constantly enter the water may represent an environmental issue [136–138]. Similar issues occur in the US for the control of *I. scapularis*, where the tick populations are maintained by wild animals, such as *Peromyscus leucopus* (white-footed mouse), other rodents and birds [95]. However, the use of topical or oral acaricides is now a reality in the US to control *I. scapularis* and *A. americanum* ticks on *Odocoileus virginianus*, the white-tailed deer [139].

### Efficacy and alternatives to increase the efficacy of EOs/EOCs

#### Efficacy of EOs/EOCs for on-host tick control

Regarding the efficacy of trials using hosts, a worrisome aspect in the review of the selected articles was the lack of information about how the tick counts were performed, the stages of ticks counted and the calculations of efficacy or mortality. Thus, we found different efficacy values without standardization of the articles, thus hampering comparison (Tables 1, 2). Such a scenario highlights that studies using EOs/EOCs have not typically followed international guidelines for evaluating the efficacy of acaricides, hence lacking standardization.

The most recent antiparasitic guidelines for acaricide registration require studies in pen facilities or fields, using both treated and control groups (untreated), with a preferred efficacy ≥ 90% [118]. Andreotti et al. [140] and Agnolin et al. [141] have reported results indicating an efficacy ≥ 90% in studies with cattle involving experiments that followed aspects mentioned in the most current guidelines [118] for efficacy verification (Table 1). The first study tested a pour-on formulation based on *T. minuta* EO at 20% on experimentally infested cattle (pen study) and obtained an efficacy of ≈100% after 13 days post-treatment (DPT). The second study performed a trial in naturally infested cattle with systematics tick counts (field study). The formulation of *Cymbopogon winterianus* EO at 8.6% was applied on cattle using a backpack sprayer. After 21 DPT, the efficacy was ≈90%. In contrast, Klafke et al. [142] tested a commercial spray with rosemary oil 10% (EO), geraniol 5% (EOC) and peppermint oil 2% (EO) (Essentria® IC-3, 6.5%) on experimentally infested cattle and observed an efficacy of 70%, 21 DPT.

Some works evaluated the treatment efficacy of EOs and EOCs in ticks infesting cattle differently than the guidelines [118], without an untreated control group. In these studies, the number of ticks was counted before and after treatment. In addition, some studies evaluated efficacy by analyzing the biology of recovered females after treating the cattle. These studies evaluated the biological parameters of engorged females in the laboratory to reach an efficacy value according to tick weight, egg mass weight and hatchability larval. Among the studies selected, Santos et al. [126], Arafa et al. [59] and Ibrahium et al. [143] accessed efficacy and found results > 70% and significantly lower tick counts after treatment (Table 1).

Santos et al. [126] used a cinnamon EO in three forms: pure oil (5%), nanocapsules (0.5%) and nanoemulsion (0.5%). Four cows from each group were sprayed with 50 mL of the tested formulation, and the test was performed on naturally infested cattle (Table 1). Animals sprayed with pure and nanoencapsulated cinnamon oil had significantly fewer ticks on days 1 and 4 post-treatment and were free of ticks on day 20 post-treatment. Engorged females collected 24 h after treatment had impaired oviposition and larval hatching, with treatment efficacy of 90, 100 and 63% to pure oil, nanocapsules and nanoemulsion, respectively.

Arafa et al. [59] tested deltamethrin, deltamethrin + thymol (EOC) and deltamethrin + thymol (EOC) + eucalyptus 5% (EO) against *R. annulatus*. Naturally infested cows from each group were sprayed with the tested formulation, and the efficacy on day 30 post-treatment was 21.6, 88.3 and 95%, respectively (Table 1). Ibrahium et al. [143] evaluated the acaricidal activity of mallow (*Pelargonium graveolens*) EO 10%. Five naturally infested cattle were sprayed with 400 mL nanoemulsion of *P. graveolens* or *P. graveolens* + sesame oil. The authors observed that both treatments reduced the tick burden by 88% and 75%, respectively, 21 DPT. From females collected 72 h after treatment, only those treated with the nanoemulsion laid no eggs.

Here, it is worth discussing the relationship between the EOs/EOCs concentrations and efficacy. Changing the dose or the concentration of the active ingredient of an acaricide formulation is a known strategy to circumvent the resistance mechanisms of ticks [91]. However, regarding EOs and EOCs, increasing the concentration can impact the feasibility of formulations due to the high cost linked to the volume (4 to 5 L) required to spray a bovine completely. Increasing concentration, coupled with applying a volume of 4 to 5 L, can also increase the chance of cattle intoxication [21]. For small ruminants, the studies used different routes of administration, making results difficult to compare (Table 2).

In studies using five experimentally infested dogs (Table 2), 100% efficacy was reported against all stages of *R. sanguineus* s.l. evaluated 24 h after spraying *T. minuta* EO at 20% [130]. The use of a commercial product containing a mixture of garlic (2.5%), allicin (0.05%) and rapeseed (8%) EOs resulted in a treatment efficacy of 75–99% from the first to the third oral dose and a preventive efficacy of 100% against experimental infestation of *R. sanguineus* s.l. in 10 dogs [131]. Monteiro et al. [60] used a nanoemulsion containing thymol and eugenol EOC (5 mg/mL) sprayed on five experimentally infested dogs and observed a lower number of larvae, but not nymphs and adults, 3 DPT. However, there was an 85% reduction in the offspring (eggs and larvae) of engorged female *R. sanguineus* s.l. recovered from the treated dogs. In addition, the engorged larvae and nymphs recovered from the treated groups did not molt. Differently from cattle, the volume of application per dog is much lower, increasing economic viability and allowing the use of higher product concentrations for the treatment of these animals.

Overall, experimental studies have indicated that immature ticks (larvae and nymphs) are more susceptible to EOs and EOCs than adults (Table 2) [60, 135]. This could be related to the features of the cuticle in immature and adult tick stages [144, 145], although this has not been properly assessed. The studies with goats and sheep cannot be considered for discussion because of the lack of information.

#### Efficacy of EOs/EOCs for tick control in the environment

Table 3 shows the details of the studies using EOs/EOCs in the environment, such as tick stages, active compounds, concentration, plot size and efficacy. The efficacy exceeded 90% in half of the studies evaluated [146–149]. However, in some studies efficacy dropped over the evaluation period, requiring new applications to maintain tick suppression in the studied areas [146, 147, 149, 150]. The low persistence and consequently decreased control rate of EOs/EOCs may be linked to their high volatility [36, 61], which can be corrected by using encapsulated formulations [151]. For instance, Dolan et al. [150] observed that using a formulation with the concentration of 2% nootkatone EOC, applied by a high-pressure sprayer, was as effective as a formulation containing 5%, applied by a backpack pump. These authors also noted that a formulation using nanotechnology increased the effectiveness of nootkatone EOC. Improvements in the formulation process, such as the nanoemulsion used by Dolan et al. [150] and the encapsulation in lignin used by Bharadwaj et al. [147], allowed the use of lower concentrations and increased the efficacy period of the product against *I. scapularis* and *A. americanum*.

In addition, combinations between EOs and EOCs can maximize efficacy, as demonstrated by Vale et al. [152]. Under laboratory conditions, these researchers found that binary combinations of thymol, carvacrol and eugenol EOCs showed synergistic effects against *A. sculptum*, allowing increased efficacy with the use of lower concentrations, in addition to reducing the costs. In the field, they observed that the combination of thymol (5.0 mg/mL) + eugenol (5.0 mg/mL) EOCs resulted in 63% efficacy, while the combination of carvacrol (5.0 mg/mL) + eugenol (5.0 mg/mL) EOCs presented 42% efficacy.

### Alternatives to increase the efficacy of EOs and EOCs

#### Nanotechnology

Six of the reviewed articles used nanotechnology to create formulations with EOs/EOCs (Tables 1, 2, 3). Using nanotechnology for formulation development is known to increase efficacy results and allow the use of lower concentrations of EOs and EOCs, which also increases the economic feasibility of the development of these biopesticides [65].

For example, against *R. microplus*, three studies demonstrated differences in the treatment efficacy when using nanotechnology (Table 1). By using tea tree (*Melaleuca alternifolia*) oil in nanocapsules (0.75%) and in its pure form (5%), Boito et al. [125] observed a control reduction of 34.5 and 0%, respectively, on tick reproductive biology, evidencing that nanoencapsulation increased efficacy. Santos et al. [126] found similar results using *Cinnamomum* sp. EO nanoencapsulated (0.5%), in nanoemulsion (0.5%) and its pure form (5%), with control reductions on *R. microplus* reproductive biology of 100%, 63.5% and 90.5%, respectively. Ibrahium et al. [143] verified that the nanoemulsion of *P. graveolens* EO was better than the association of *P. graveolens* EO with sesame oil. Only the females treated with the nanoemulsion did not oviposit. Against *R. sanguineus* s.l., Monteiro et al. [60] used nanoemulsion with thymol and eugenol (5 + 5 mg/mL = 1% active) EOCs on dogs, with larval reduction and 85% efficacy on the reproductive biology of engorged females (Table 2).

Dolan et al. [156] applied nootkatone EOC in the environment (shrub and litter layer) to suppress *A. americanum* and *I. scapularis* nymphs and observed a higher reduction of the ticks with the use of a nanoemulsion compared with a simple emulsion, 28 DPT (Table 3). Bharadwaj et al. [153] observed that a formulation of lignin-encapsulated nootkatone, applied in a residential lawn perimeter, resulted in 100% of control for *I. scapularis* nymphs for 56 days, whereas an emulsifiable formulation of nootkatone showed 100% control of the nymphs for only 7 days.

#### EOs/EOCs combined with synthetic acaricides

The association of EOs/EOCs with synthetic acaricides is another possibility to improve efficacy (Table 1), with an approach to find a synergistic effect as demonstrated in laboratory assays using eucalyptus EO + thymol EOC + deltamethrin against *R. annulatus*, and thymol EOC + cypermethrin, (*E*)-cinnamaldehyde EOC + amitraz and (*E*)-cinnamaldehyde EOC + chlorfenvinphos against *R. microplus* [21, 57, 59]. The Brazilian market already has formulations of commercial acaricides containing pyrethroids and organophosphates associated with terpenes (citronellal and geraniol EOCs) or piperonyl butoxide [153], a semisynthetic derivative of safrole EO, which is a phenylpropanoid found in plants of the genus *Piper* [154].

Initial data with *R. microplus* indicate that there is no cross-resistance between synthetic acaricides and EOs/EOCs [92, 155, 156]. In other words, tick populations resistant to commercial acaricides are not resistant to EOs/EOCs. Thus, combinations of synthetic acaricides with EOs/EOCs are an interesting alternative to be further investigated [92, 155, 156].

Two field studies in this review associated EOs/EOCs with synthetic acaricides (Table 1) [21, 59]. Arafa et al. [59] used a combination of eucalyptus EO + thymol EOC + deltamethrin that resulted in 95% efficacy in controlling *R. annulatus* infestations in cattle; in turn, when using only deltamethrin, the effectiveness was 21%. Gonzaga et al. [21] evaluated a combination of (*E*)-cinnamaldehyde EOC + amitraz against *R. microplus* in cattle. However, a few minutes after treatment the bovines showed intoxication signs, and the experiment could not proceed.

### Clinical safety for hosts

Of the 23 articles using EOs/EOCs applied on animals, 65.2% evaluated at least one variable regarding the safety of the formulations for animals, such as heart and respiratory rates, rectal and eyeball temperatures, dehydration and mucous membrane coloration changes. Among these variables, no adverse changes were reported in 86.6% of the studies. However, few studies have performed more complete evaluations, including on hemogram, biochemical, clinical and dermal changes (Table 4).

**Table 4:** Clinical safety performed in 23 articles using essential oils (EOs) and essential oil compounds (EOCs) to control ticks in treated animals

Hosts	EOs/EOCs	Evaluated parameter				References
		Hemogram	Biochemistry	Clinical evaluation	Dermal evaluation	
Cattle	*C. citratus* e C*. nardus*	…	…	…	…	Chungsamarnyart and Jiwaginda [183]
Rabbits	*O. suave*	…	…	…	No adverse reaction	Mwangi et al. [135]
Cattle	*C. winteranius Jowitt*	…	No change	…	…	Martins and González [124]
Goats	*C. citratus* and* C. nardus*	…	…	No adverse reaction	No adverse reaction	John et al. [132]
Holstein cattle	*C. nardus*	No change	…	…	…	Agnolin et al. [157]
Holstein cattle	*C. nardus*	…	…	…	…	Agnolin et al. [184]
Sheep	*Th. trilobata*	…	…	…	…	Peebles et al. [134]
Holstein cattle	*Co. citriodora*	…	…	No adverse reaction	No adverse reaction	Olivo et al. [159]
Holstein cattle	*T. minuta*	…	…	…	No adverse reaction	Andreotti et al. [140]
Holstein cattle	*C. winterianus*	…	…	No adverse reaction	No adverse reaction	Agnolin et al. [141]
Holstein cattle	*Co. citriodora* and *Co. citriodora* modified	…	…	…	…	Chagas et al. [185]
Goats	*Ch. ambrosioides*	…	…	No adverse reaction	…	Kouam et al. [133]
Dogs (mixed breeds)	*T. minuta*	…	…	…	…	Silva et al. [130]
Holstein cattle	*M. alternifolia*	…	…	…	…	Boito et al. [125]
Holstein cattle	*Cinnamomum* sp.	…	…	…	…	Santos et al. [126]
Holstein cattle (calves)	Eugenol	…	…	No adverse reaction	No adverse reaction	Valente et al. [158]
Dogs (mixed breeds)	Lacecca® (*A. sativum*, + Allicin + *B. napus)*	No change	No change	No adverse reaction	No adverse reaction	Amer and Amer [131]
Baladi-Holstein cattle (cross breed)	Thymol and *E. globulus* combined with deltamethrin	…	No change	No adverse reaction	Allergic reaction in an animal	Arafa et al. [59]
Cocker Spaniel English dogs	Thymol + eugenol	No change	No change	No adverse reaction	No adverse reaction	Monteiro et al. [60]
Aberdeen-Angus cattle	Essentria® IC-3 (rosemary oil 10%, geraniol 5% and peppermint oil 2%)	…	…	No adverse reaction	…	Klafke et al. [142]
Simmental cattle	(*E*)-cinnamaldehyde	…	…	Sialorrhea and muscle tremors	No adverse reaction	Gonzaga et al. [21]
Girolando (Gyr × Holstein) cattle	*L. sidoides*	…	…	…	…	Pereira et al. [180]
Native breed cattle	*P. graveolens* L	…	…	No adverse reaction	No adverse reaction	Ibrahium et al. [143]

Abbreviations of the genera of plants: *Cymbopogon* (C.)*, Ocimum (O.), Thelechitonia (Th.), Corymbia (Co.), Tagetes (T.), Chenopodium (Ch.), Melaleuca (M.), Allium (A.), Brassica (B.), Eucalyptus (E.), Lippia (L.), Pelargonium (P.)*

Evaluation not carried out

Clinical evaluation: Heart and respiratory rates, rectal and eyeball temperatures, dehydration and mucous membrane coloration changes

For cattle, three studies verified the hemogram. Hemogram was performed in a study using *C. nardus* EO at 4% [157], while biochemical evaluation was also performed in studies including *C. winteranius* EO pure and at 10% [124] and eucalyptus (*Eucalyptus globulus*) EO at 5% + thymol EOC + deltamethrin [59] (Tables 1 and 4). The clinical evaluation did not describe the clinical parameters evaluated in the studies using cattle by Valente et al. [158], Arafa et al. [59] and Klafke et al. [142]. Heart and respiratory rate values and eyeball temperature were evaluated in the studies with Holstein cattle by Olivo et al. [159] and Agnolin et al. [141]. An adverse reaction was perceived only in the study of Gonzaga et al. [21], in which the Simmental cattle treated with (*E*)-cinnamaldehyde EOC at 0.1% showed sialorrhea and muscle tremors. Arafa et al. [59] observed a dermal alteration in a bovine sprayed with 1 mL/L of thymol EOC, with precipitation of thymol crystals appearing on the animal’s skin, causing local irritation. Dermal evaluation, in cases of toxicity, may reveal allergic dermatitis and urticarial lesions in addition to reddening and warmth of the skin as a function of vasodilation caused by rubefacient agents, as observed for some EOs [160, 161].

For dogs, hemogram and biochemical analyses were performed before and after the treatment using a commercial product based on allicin and EOs of garlic (*Allium sativum*) and rapeseed (*Brassica napus*) [131] in addition to a formulation containing a combination of thymol with eugenol EOCs [60] (Tables 2 and 4). There was no change in the blood count and biochemistry parameters of treated dogs in these studies. Monteiro et al. [60] evaluated the rectal temperature, hydration, heart and respiratory rates as well as mucous membrane coloration and general physical condition of English Cocker Spaniel dogs treated with a nanoemulsion containing thymol (5.0 mg/mL) + eugenol (5.0 mg/mL) EOCs. No dermal alterations were observed, a fact that may be related to the stability presented by the formulation, preventing the precipitation of thymol EOC. Another factor that might explain the absence of dermal reactions is the presence of eugenol EOC in the formulation. Data have shown that the presence of eugenol EOC minimizes or even prevents skin reactions caused by other compounds also present in EOs [115, 162].

For small ruminants, clinical signs and clinical pathological abnormalities have not been evaluated (Tables 2 and 4). Kouam et al. [133] only reported that goats treated with the *C. ambrosioides* EO soap foam did not change their behavior. However, the authors did not present details regarding this evaluation.

It is important that future studies properly assess the safety of EOs and EOCs in addition to the evaluation of efficacy. A standardized evaluation of clinical signs and clinicopathological abnormalities would allow a proper comparison of different treatment regimens in addition to providing more accurate data regarding EOs and EOCs safety.

### Non-target organisms: residues and toxicity

The EOs possess numerous biological activities and are effective against various pests, having little or no toxicity against non-target species, as demonstrated in the EOs of species like fennel (*Foeniculum vulgare*), stevia (*Stevia rebaudiana*) and cinnamon (*Cinnamomum cassia*) [163–166]. In this review, toxicity effects on non-target species or description of residues in the environment were evaluated in only 25% of field and semi-field studies conducted in the environment (Table 3) [147, 167]. There were reports of decreased numbers of non-target arthropods of the orders Coleoptera, Hymenoptera and Collembola 1 week after the application of a product based on rosemary and peppermint EOs + geraniol EOC [167]. Phytotoxicity of products based on rosemary and peppermint EOs + geraniol EOC and emulsifiable nootkatone EOC was also reported. However, the authors mention that this phytotoxicity was reversed days after application [147, 167].

These assessments are important, especially in studies in the environment, as there is evidence of toxicity of EOs from bushy mat grass (*Lippia alba*), *L*. *gracilis*, spiced rosemary (*L*. *sidoides*), wild mint (*Mentha arvensis*), peppermint (*M*. *piperita*), clove basil (*Ocimum gratissimum*), pepper plants (*Piper aduncum* and *P*. *callosum*) and the hydrolate of common wormwood (*Artemisia absinthium*—a byproduct of its EO) on micro-crustaceans, plant seeds, algae and nematodes [168–170]. One possibility to decrease and even avoid phytotoxicity is encapsulation with lignin, as used by Bharadwaj et al. [147]. These alternatives such as the use of nanotechnology can reduce potential risks to animals and non-target organisms [171].

## Animal health industry point of view

The industry plays a fundamental role in the development of new acaricides, of either chemical origin or not, by translating research into tangible products. The global animal health sector was valued in 2021 at $38.3 billion, and the parasiticide sales corresponded to the biggest chunk of the market, accounting for 34.1% of the revenues, followed by vaccines (28.5%), other products (22.2%) and antimicrobials (15.2%) [172]. In Brazil, the animal health sector moved approximately $2 billion, with parasiticides representing about 25% of the revenues [153]. Overall, this highlights the importance of parasites for the animal health sector globally.

The commercial attractiveness of the parasiticide segment attracts significant investment for the development of new solutions for parasite control annually [173]. However, sales potential is not the only motivating factor for a new project; additional financial indicators, like expected profitability and net present value (NPV), also play an important role in the decision process. Other aspects to be considered before starting a project for the development of a new antiparasitic are the strategic fit with the overall company strategy, technical feasibility and legal certainty (animal health industry personal communication). The development of a new product results from a complex, long-term, expensive and multidisciplinary process. A project team is required, usually composed of a project leader and representatives of the following areas: marketing, manufacturing and controls guidance, regulatory affairs, finance, clinical studies and supply. Typically, the development process of an innovative product (based on a new mode-of-action active pharmaceutical ingredient) takes 10–15 years to be completed and costs around 30–40 million euros. The project team is responsible for the planning and execution of initiatives to ensure the product meets all regulatory requirements (quality, efficacy and animal/human/environment safety) and is granted official market authorization (animal health industry personal communication).

In the last 30 years, the animal health industry witnessed drastic changes concerning the development of new molecules or innovative products for parasite control. Technology advances (e.g. in structural biology, computational chemistry, structure-based drug design, genomics and proteomics) have accelerated the selection of new parasiticide candidates [173]. However, there is an increasing demand for eco-friendly (‘green’) products that reduce or eliminate parasites, without compromising safety or cost efficiency [173]. Indeed, new products are required to be safe not only for the target species but also for non-target species and the environment. According to the Food and Drugs Administration (FDA), there was a 2.7% increase in investments in research and development (R&D) by the industry from 1989 ($ 604 million) to 2017 ($ 1.1 billion). However, during the same period, the number of approvals of new molecules declined by 3.6% [174]. This highlights that, despite technological advances, the marketing authorization requirements for parasiticides have become more stringent [175].

The use of natural products to control ticks is a current trend since issues of sustainability, one health and animal welfare are increasingly present and permeation in society is necessary as soon as possible, whether in animal production or for companion animals (animal health industry personal communication). The availability of EOs and EOCs based products on the veterinary market is currently limited. This may be related to several factors, including the lack of randomized clinical trials conducted according to current regulatory requirements for marketing authorization of products, whose efficacy has been demonstrated in laboratory studies only. In this regard, registration of new products is a lengthy process, and efficacy requirements may be excessively high. For example, the current Brazilian legislation for licensing anti-parasitic products for veterinary use dates to 1997 [119]. To be approved for the control of *R. microplus*, a product must present an average efficacy of at least 95% on 23 DPT in pen studies and on 7 and 14 DPT in field studies [119].

The in vitro acaricidal efficacy of EOs and EOCs is often promising. However, in vitro results are not always observed in field trials, especially in terms of persistent efficacy [176]. Perhaps, updated legislation with less stringent efficacy requirements could accelerate the marketing authorization of new products, including EOs and EOCs based products, which could be used for integrated tick management. There is a need for a broad discussion on the harmonization and efficacy requirements for these products, which should involve researchers, government agencies and industry. Another challenge related to the registration of EOs or EOCs based products is safety. While these products are usually believed to be eco-friendly, not all EOs and EOCs are innocuous to animals, harmless to the environment or leave no residues in meat and milk. Prolonged exposure to high concentrations of certain EOs can have deleterious effects on the behavior, health and welfare of the host [21, 147, 167, 177].

Other practical issues are linked to the supply, standardization and economic viability of EOs and EOCs. A reliable supply of affordable and standardized raw materials in sufficient amounts to meet the market demand can be a challenge for a product based on EOs or EOCs [178]. The secondary metabolism of a plant and EOs composition is directly affected by the soil acidity and climate (heat, photoperiod and humidity) [179]. Furthermore, most commonly, the biological effect of an EO is triggered by a composition of molecules (rather than one single compound), which raises the problem of how to perform the raw material quality check while not knowing all the substances that should be quantified. Consequently, the usual quality checkpoints during and at the end of the manufacturing process could also be tricky.

Regarding economic viability, the concentrations of EOs and EOCs that present efficacy are often high. This can make the production of a tick control product for cattle unfeasible, where 1 L of product needs to be diluted in large volumes of water (> 400 L) to allow treatment of multiple animals. For example, Pereira et al. [180], in a field study with *R. microplus*-infested cattle, observed an average effectiveness of 50%, reaching 63% on day 21, using *L. sidoides* EO at a concentration of 1% (10,000 ppm). This concentration is much higher than that found in commercially available spray products for control on cattle (generally > 1000 ppm) [21]. In tick control on dogs, a smaller amount of product is necessary to treat the animals, added to the fact that products for dogs are generally already available ready to use, without the need for dilution in large volumes of water.

Studies with structural modifications of EOCs [154, 181], development of formulations with nanotechnology [60, 125, 126] and combinations of botanical compounds [21, 59, 156, 182] with synthetic acaricides, as previously discussed, may be alternatives to solve these challenges, allowing the development of new technologies to control ticks on different animal species. In sum, despite the barriers mentioned, the animal health industry understands that the exploration of EOs and EOCs as a veterinary antiparasitic is an exciting endeavor (animal health industry personal communication).

## Conclusions

This article provides a comprehensive review of the use of EOs and EOCs to reduce tick infestations on hosts and in the environment. Despite the research advances in this field of research, we conclude that there are still several research gaps and the urgent need for more randomized clinical trials that could allow the evaluation of the efficacy of EOs and EOCs based products for the control of ticks under field conditions. Future research should also consider the following critical points: (i) characterization of the EOs or description of the source, lot number and purity degree of the EOCs; (ii) standardization of the methods used to evaluate the efficacy of EOs and EOCs, following international guidelines (e.g. WAAVP guidelines) and national/regional regulatory agencies; (iii) formulation development, especially using nanotechnology and encapsulation, allowing to reduce the volatility of EOs and EOCs, which may increase efficacy and safety; (iv) evaluation of EOs and EOCs safety for target and non-target animals and the environment. Finally, (v) studies assessing the efficacy of synthetic acaricides already in the market in combination with EOs or EOCs could provide valuable information on their synergistic activity against ticks and usefulness from an integrated tick management perspective.

### Supplementary Information

**Additional file 1.** Botanical species used to extract essential oils and compounds present in essential oils that were used in field and semi-field studies to control ticks.

## Data Availability

The data that support the findings of this study are available in the references information of this article.
