# Supplementary material for: Essential oils and isolated compounds for tick control: advances beyond the laboratory
Source: Parasit Vectors. 2023 Nov 14;16:415. doi: 10.1186/s13071-023-05969-w (PMC10647118; doi:10.1186/s13071-023-05969-w)
Supplement: Supplementary file 1 — Additional file 1. Botanical species used to extract essential oils and compounds present in essential oils that were used in field and semi-field studies to control ticks. [file 13071_2023_5969_MOESM1_ESM.docx]

[
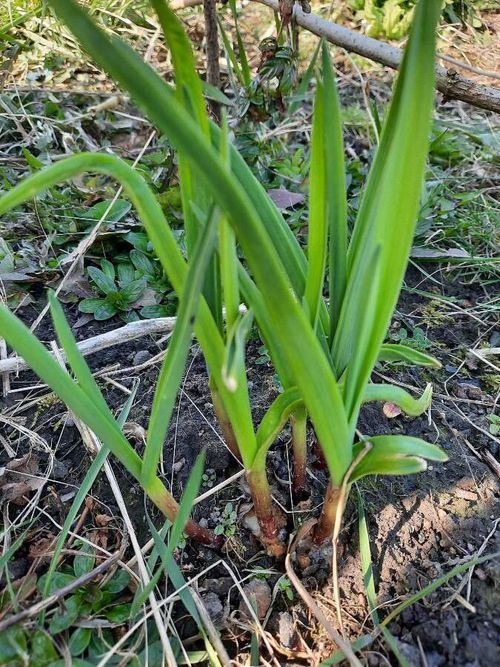
](https://www.gbif.org/occurrence/3949702475) [
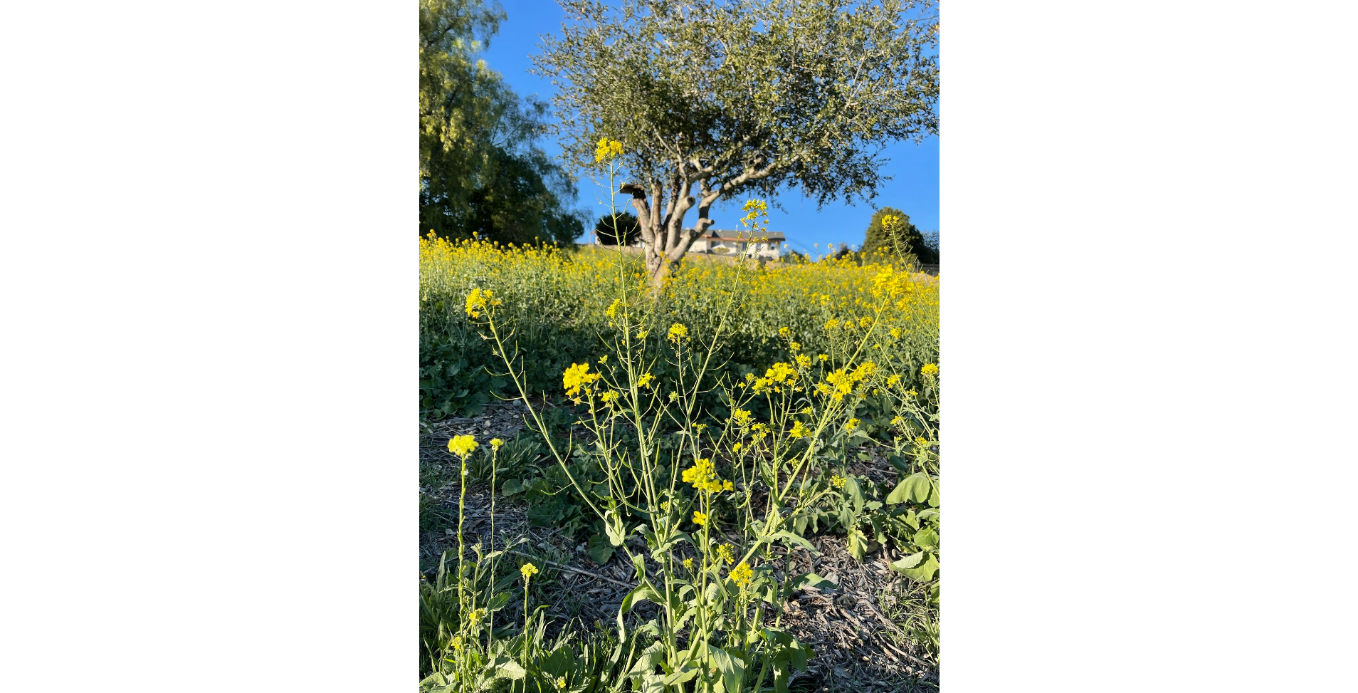
](https://www.gbif.org/occurrence/3468884067) [
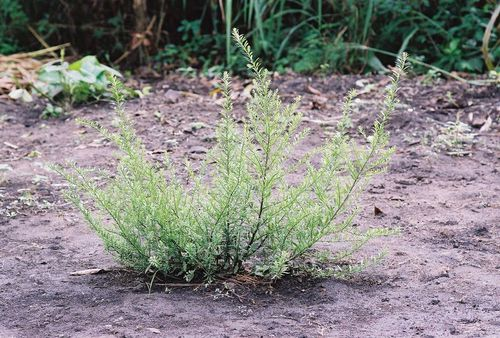
](https://www.gbif.org/occurrence/1288062096) [
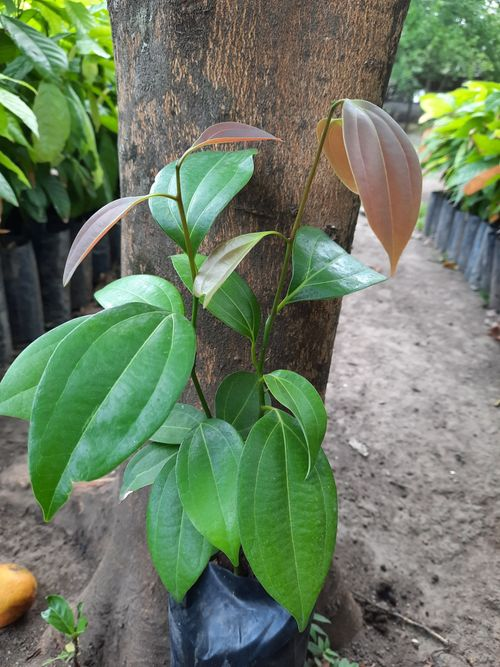
](https://www.gbif.org/occurrence/3772411698)[
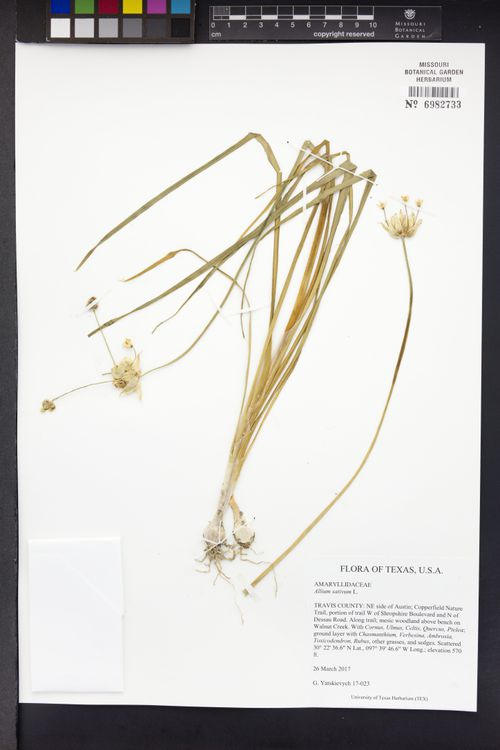
](https://www.gbif.org/occurrence/2268970445) [
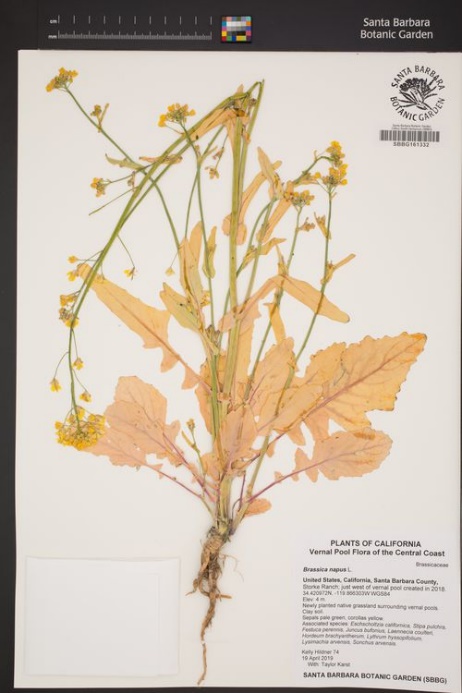
](https://www.gbif.org/occurrence/3053401861)
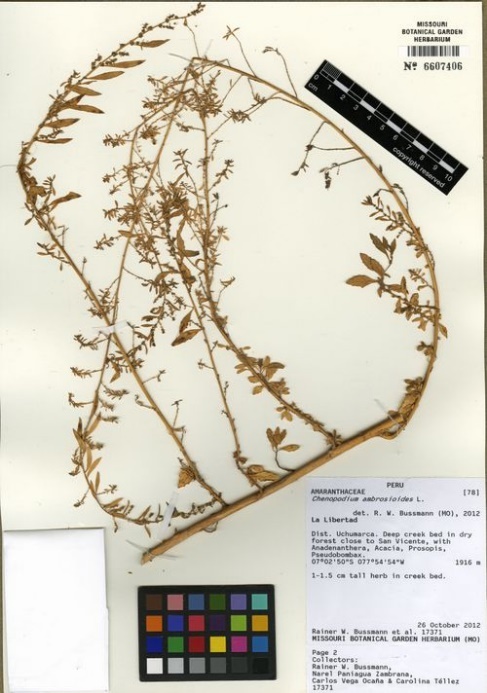
 [
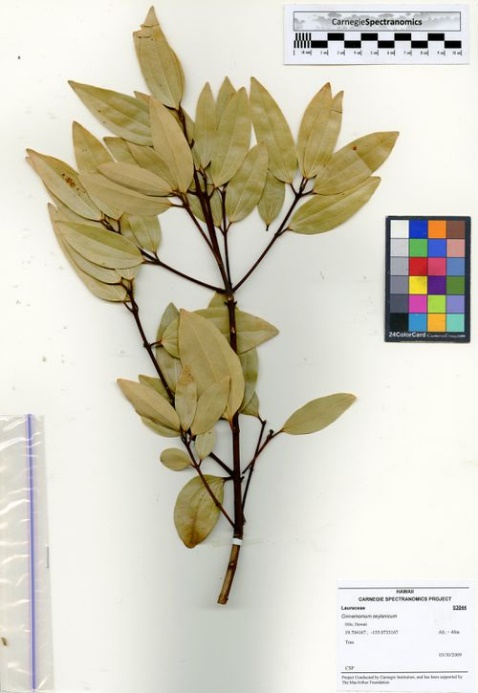
](https://www.gbif.org/occurrence/3762896331)

**A**

**B**

**C**

**D**

Supplementary figure 1 - Botanical species used for the production of essential oils that were used in field and semi-field studies for tick control. **A** - *Allium sativum* L (Amaryllidaceae); **B** - *Brassica napus* L (Brassicaceae); **C** - *Chenopodium ambrosioides* L [Accepted name: *Dysphania ambrosioides* (L.) Mosyakin & Clemants (Amaranthaceae)]; **D** - *Cinnamomum zeylanicum* Blume [Accepted name: *Cinnamomum verum* J.Presl (Lauracea)] Source: Adapted from Global Biodiversity Information Facility [187]

[
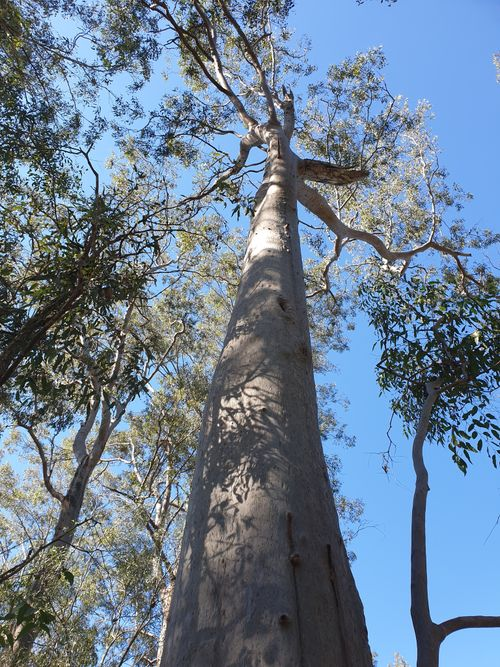
](https://www.gbif.org/occurrence/3330493235) [
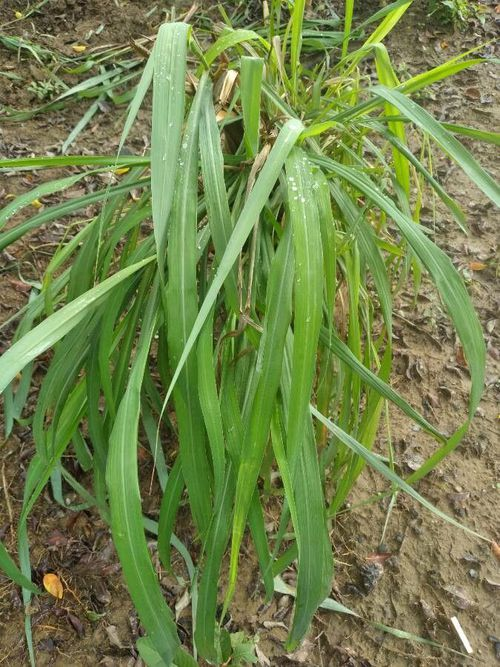
](https://www.gbif.org/occurrence/3949364803) [
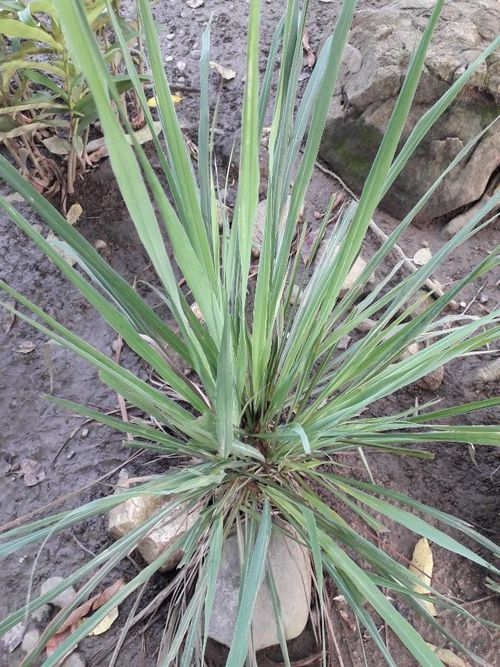
](https://www.gbif.org/occurrence/2644293491) [
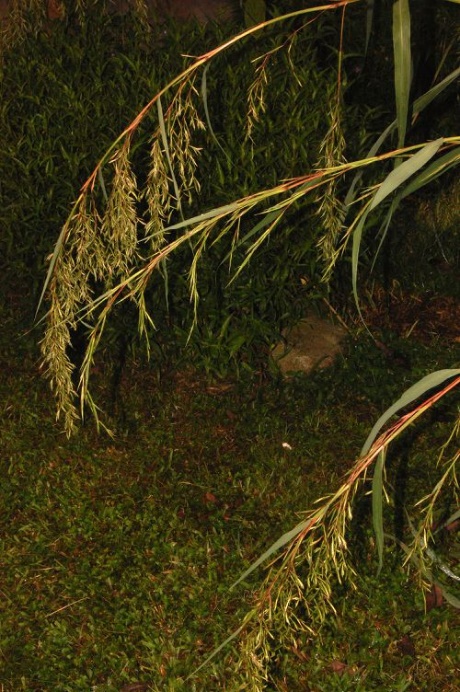
](https://www.gbif.org/occurrence/3946772779)

[
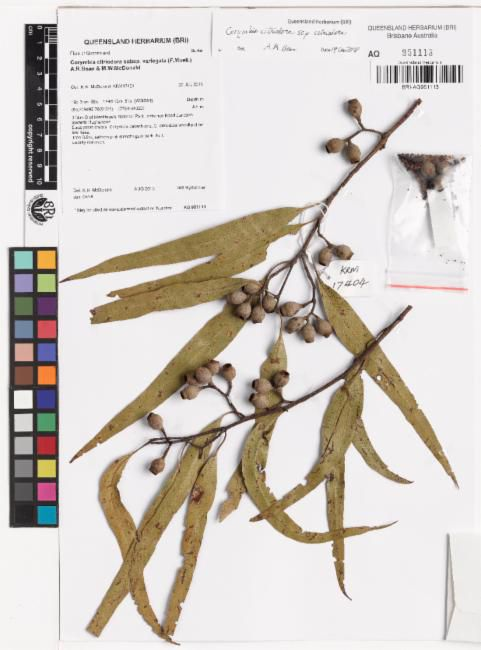
](https://www.gbif.org/occurrence/2418739569) [
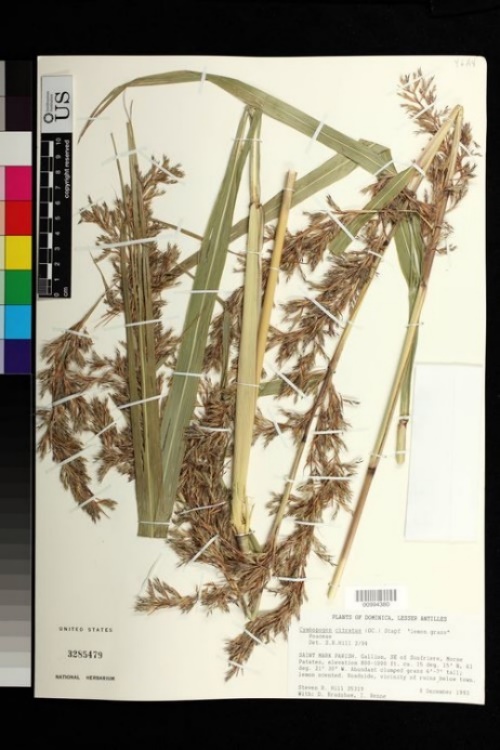
](https://www.gbif.org/occurrence/1322947064) [
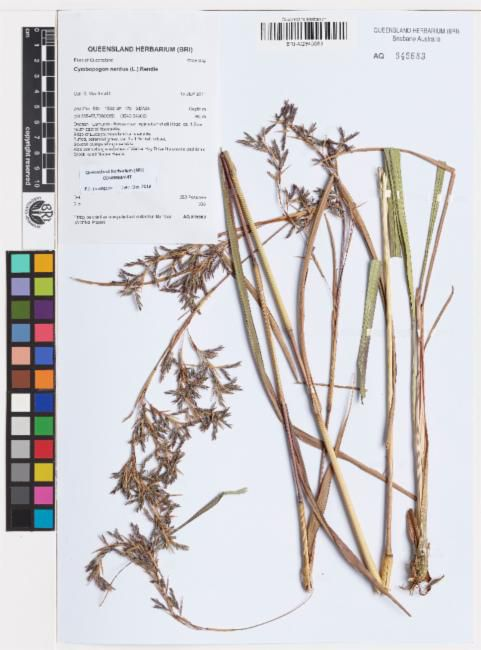
](https://www.gbif.org/occurrence/2825393946) [
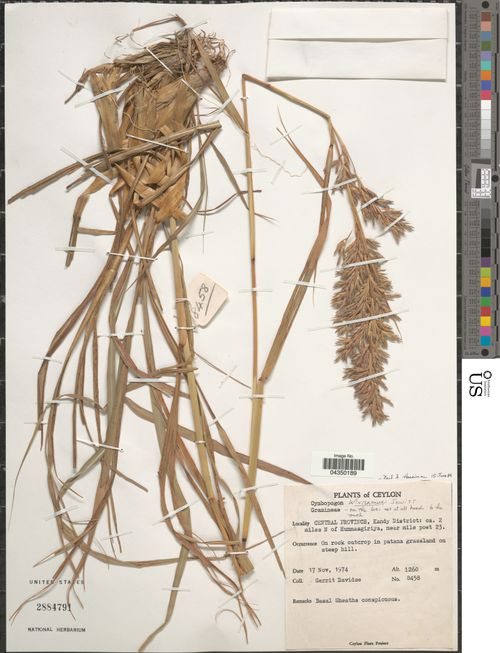
](https://www.gbif.org/occurrence/3043541418)

**A**

**B**

**C**

**D**

Supplementary figure 2 - Botanical species used for the production of essential oils that were used in field and semi-field studies for tick control. **A** - *Corymbia citriodora* (Hook.) K.D.Hill & L.A.S.Johnson (Myrtacea); **B** - *Cymbopogon citratus* (DC.) Stapf (Poaceae); **C** - *Cymbopogon nardus* (L.) Rendle (Poaceae); **D** - *Cymbopogon winterianus* Jowitt ex Bor (Poaceae) Source: Adapted from Global Biodiversity Information Facility [187]

[
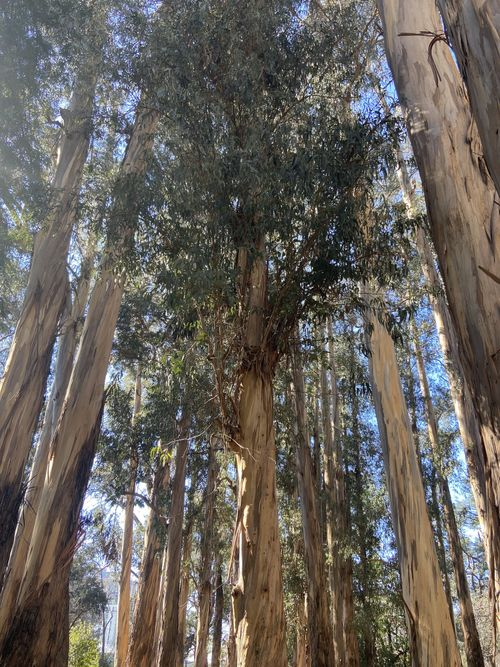
](https://www.gbif.org/occurrence/3705807585) [
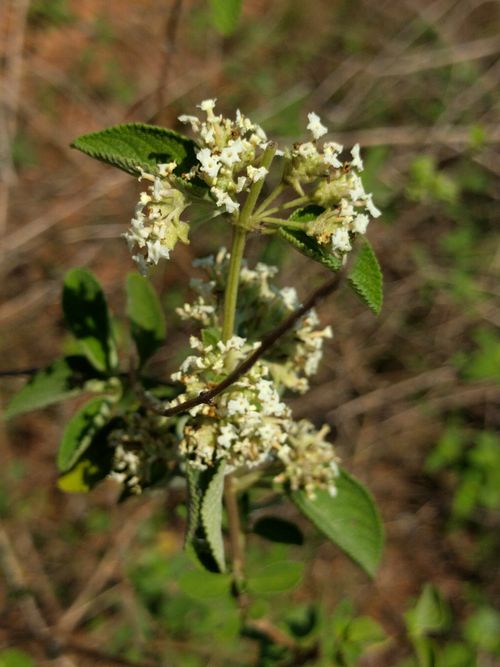
](https://www.gbif.org/occurrence/3784758330) [
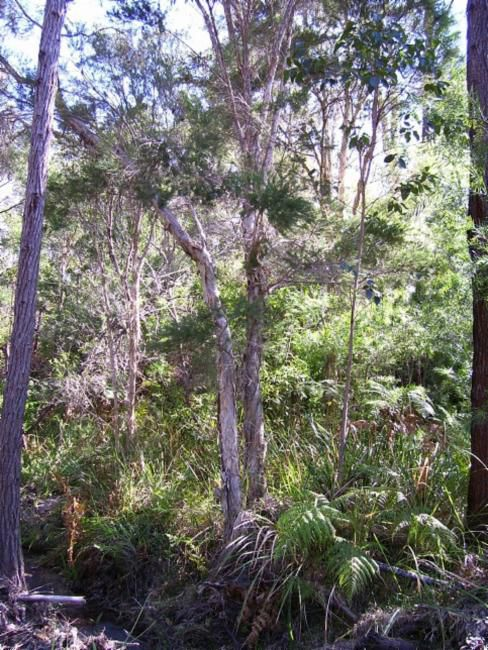
](https://www.gbif.org/occurrence/1632942275) [
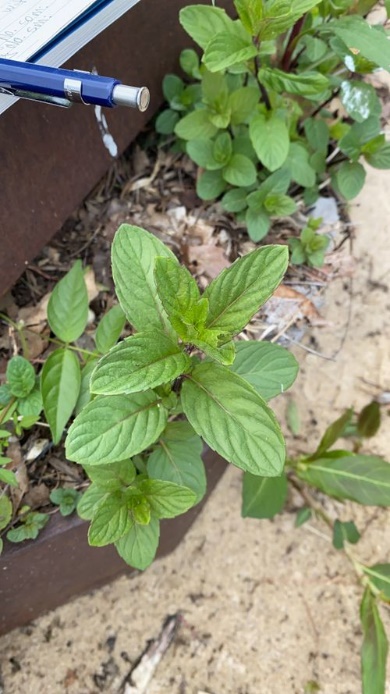
](https://www.gbif.org/occurrence/3859508650)

[
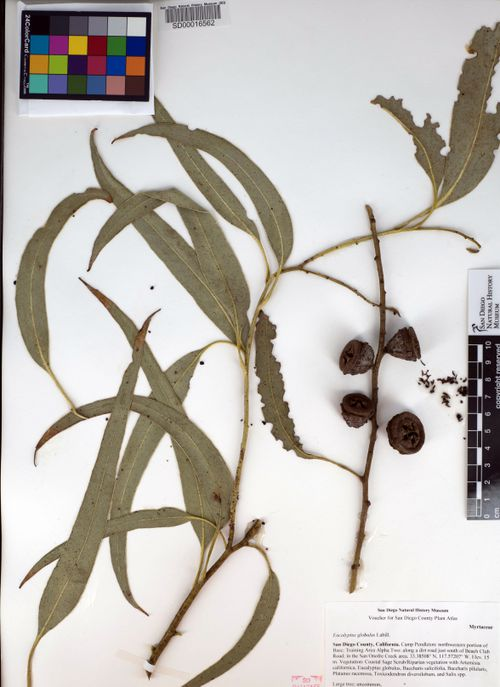
](https://www.gbif.org/occurrence/3125144336) [
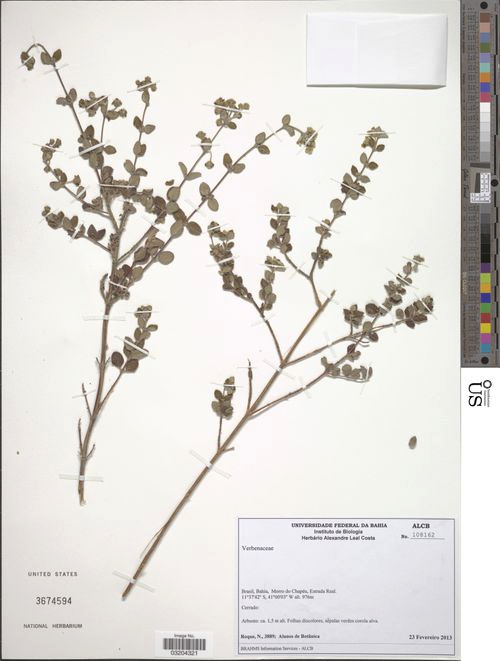
](https://www.gbif.org/occurrence/2284210631) [
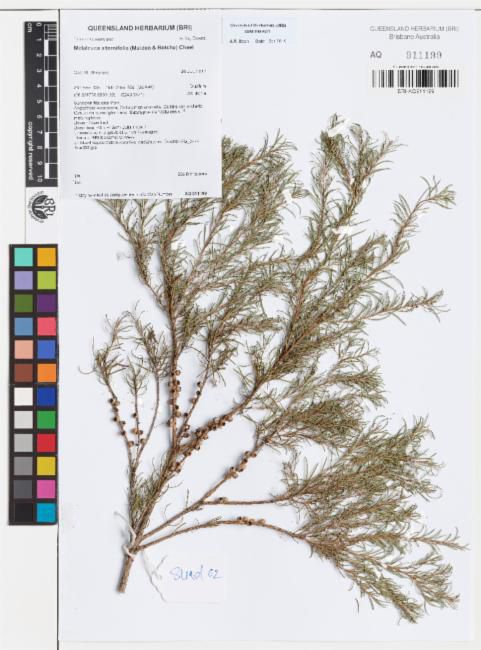
](https://www.gbif.org/occurrence/2418922104) [
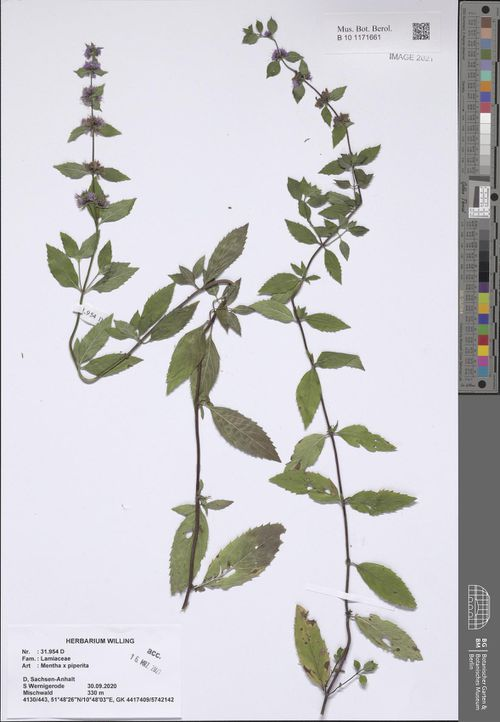
](https://www.gbif.org/occurrence/3229118382)

**A**

**B**

**C**

**D**

Supplementary figure 3 - Botanical species used for the production of essential oils that were used in field and semi-field studies for tick control. **A** - *Eucalyptus globulus* Labill (Myrtacea); **B** - *Lippia sidoides* [Accepted name: *Lippia origanoides* Kunth (Verbenaceae)]; **C** - *Melaleuca alternifolia* (Maiden & Betche) Cheel (Myrtacea); **D** - *Mentha ×piperita* L. (Lamiaceae) Source: Adapted from Global Biodiversity Information Facility [187]

[
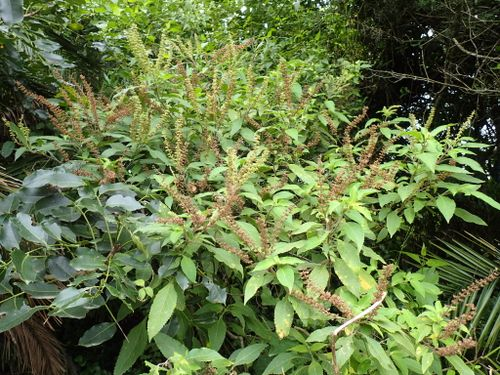
](https://www.gbif.org/occurrence/3337423442) [
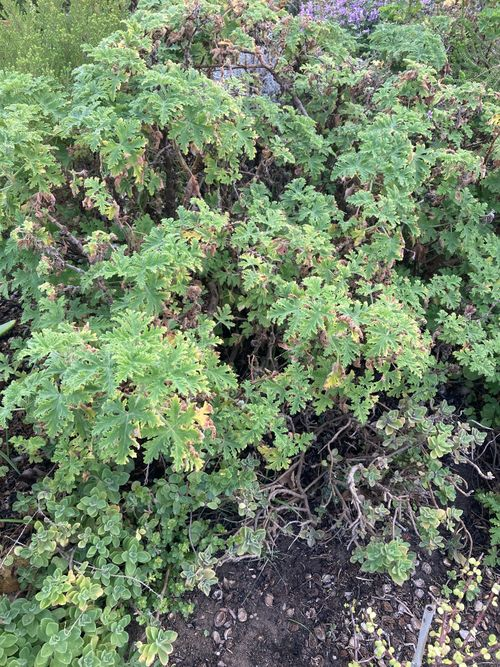
](https://www.gbif.org/occurrence/gallery?taxon_key=3116848) [
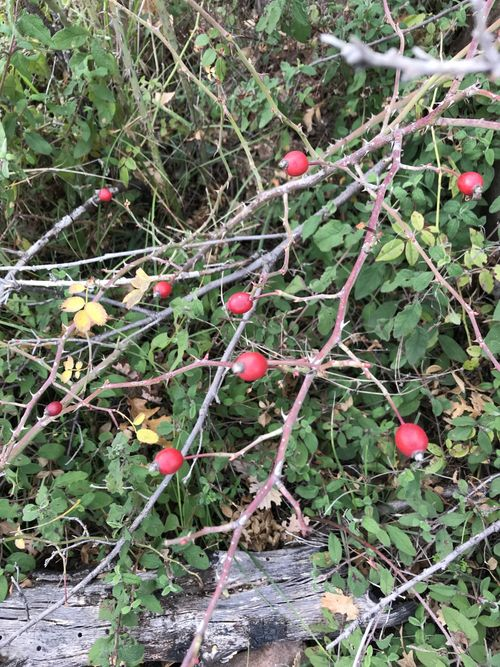
](https://www.gbif.org/occurrence/3456315026) [
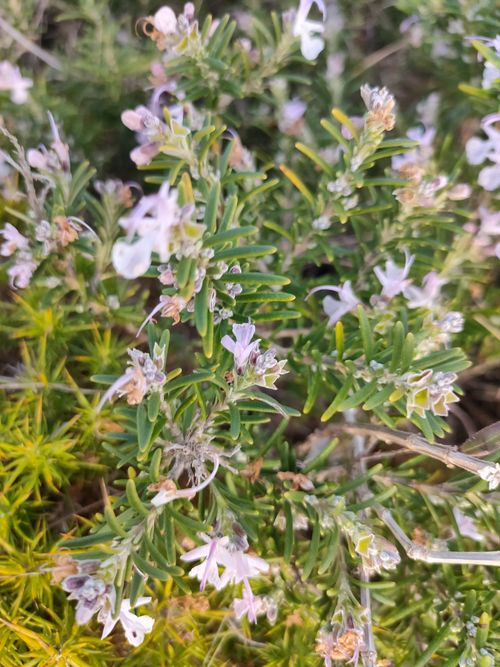
](https://www.gbif.org/occurrence/3456651366)

[
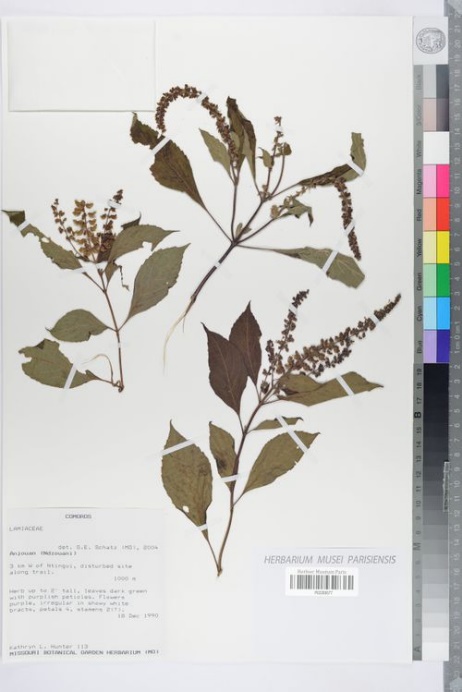
](https://www.gbif.org/occurrence/1702502437) [
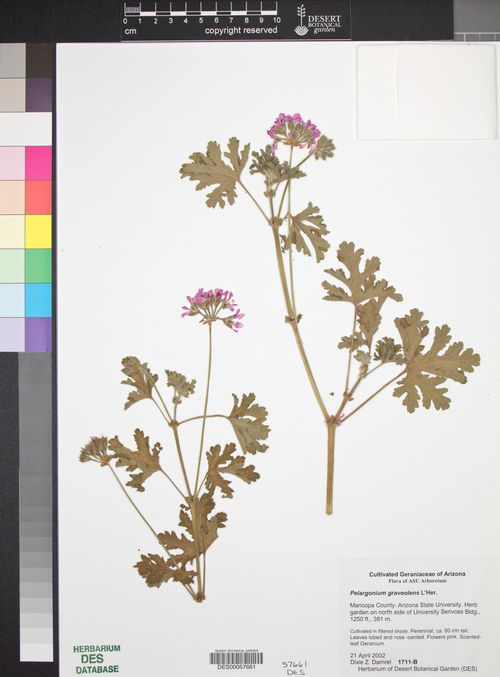
](https://www.gbif.org/occurrence/gallery?taxon_key=3116848) [
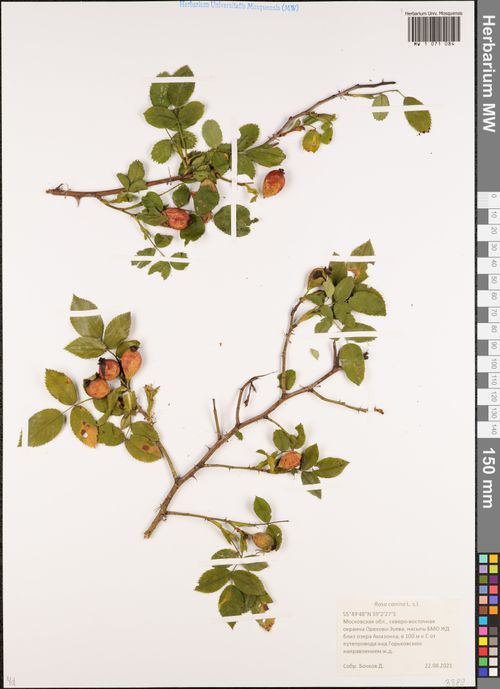
](https://www.gbif.org/occurrence/3424021629) [
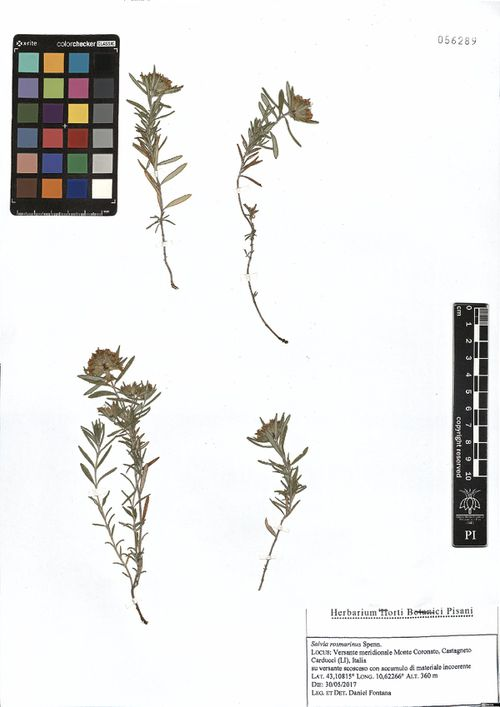
](https://www.gbif.org/occurrence/3749715419)

**A**

**B**

**C**

**D**

Supplementary figure 4 - Botanical species used for the production of essential oils that were used in field and semi-field studies for tick control. **A** - *Ocimum suave* Willd [Accepted name: *Ocimum gratissimum* subsp. *gratissimum* L. (Lamiaceae)]; **B** - *Pelargonium graveolens* (Thunb.) L'Hér (Geraniaceae); **C** - *Rosa canina* L. (Rosaceae ); **D** - *Salvia rosmarinus* Spenn. (Lamiaceae) Source: Adapted from Global Biodiversity Information Facility [187]

[
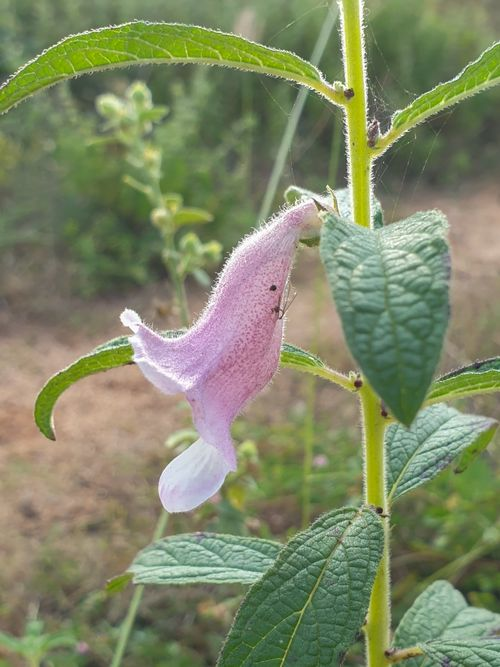
](https://www.gbif.org/occurrence/3764143874) [
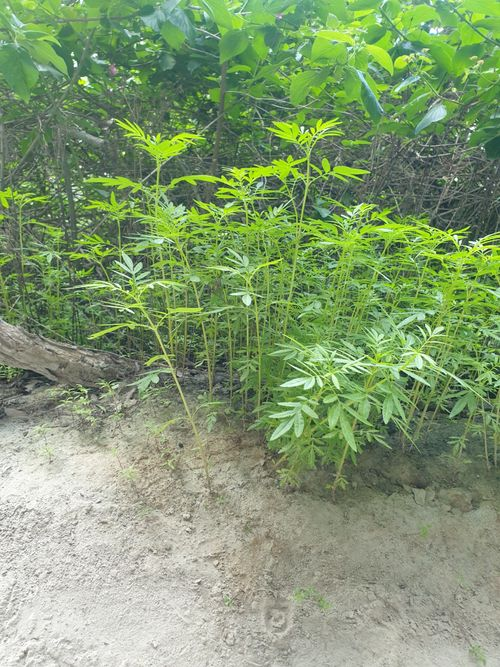
](https://www.gbif.org/occurrence/3456972154) [
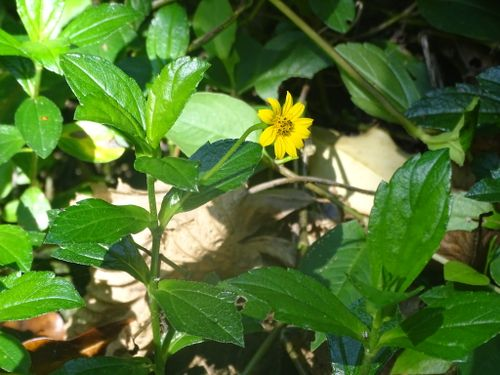
](https://www.gbif.org/occurrence/3466375212)

[
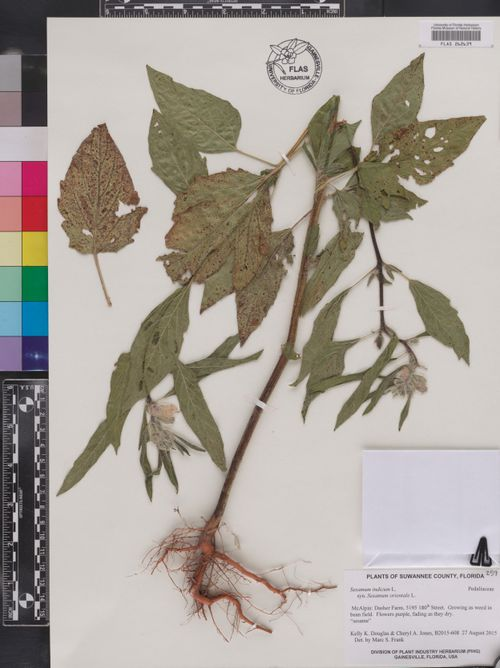
](https://www.gbif.org/occurrence/2005422597) [
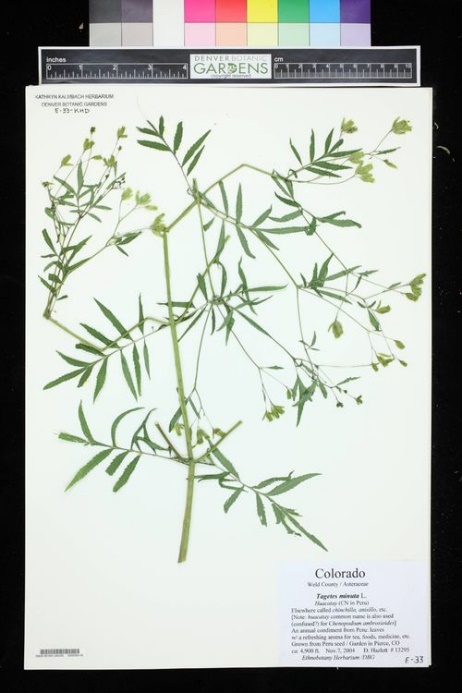
](https://www.gbif.org/occurrence/2242416216) [
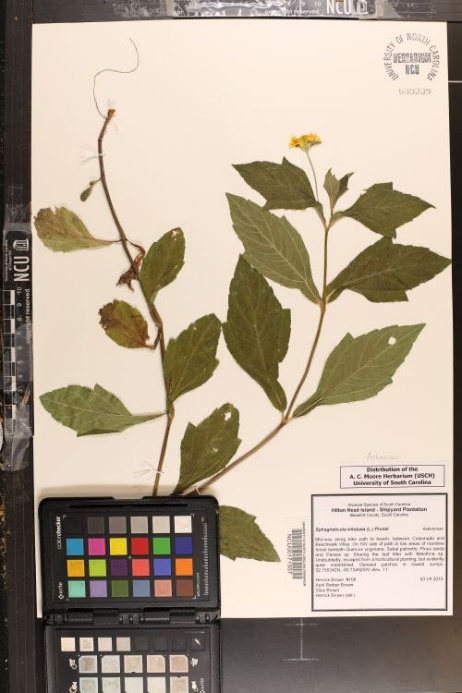
](https://www.gbif.org/occurrence/2234625469)

**A**

**B**

**C**

Supplementary figure 5 - Botanical species used for the production of essential oils that were used in field and semi-field studies for tick control. **A** - *Sesamum indicum* L. (Pedaliaceae); **B** - *Tagetes minuta* L (Asteracea); **C** - *Thelechitonia trilobata* (L.) H.Rob. & Cuatrec. [Accepted name: *Sphagneticola trilobata*(L.) Pruski (Asteracea)] Source: Adapted from Global Biodiversity Information Facility [187]


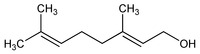

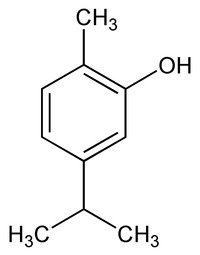

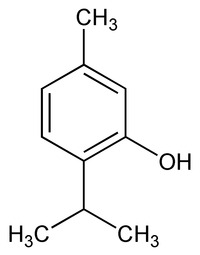

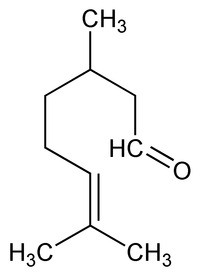


**D**

**A**

**B**

**C**


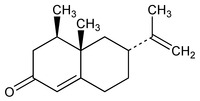

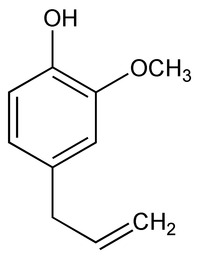

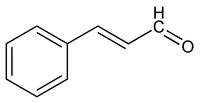


**E**

**F**

**G**

Supplementary figure 6 - Compounds present in essential oils that were used in field and semi-field studies for tick control. **A** - Carvacrol (monoterpene - phenol); **B** - Thymol (monoterpene - phenol); **C** - Geraniol (monoterpene - alcohol); **D** - Citronellal (monoterpene - aldehyde); **E** - Nootkatone (sesquiterpene - ketone); **F** - Eugenol (phenylpropanoid - phenol); **G** - (*E*)-Cinnamaldehyde (phenylpropanoid - aldehyde). Source: Adapted from Royal Society of Chemistry [188]
